# Quantitative analysis of the effects of essential oil mouthrinses on clinical plaque microbiome: a parallel-group, randomized trial

**DOI:** 10.1186/s12903-024-04365-9

**Published:** 2024-05-18

**Authors:** Kyungrok Min, Andrew J. Glowacki, Mary Lynn Bosma, James A. McGuire, Sandy Tian, Kathleen McAdoo, Alicia DelSasso, Tara Fourre, Robert J. Gambogi, Jeffery Milleman, Kimberly R. Milleman

**Affiliations:** 1 Johnson & Johnson Consumer Inc, Skillman, NJ USA; 2 Salus Research Inc, Fort Wayne, IN USA

**Keywords:** Oral Microbiome, Quantitative Microbiome, Gingivitis, Listerine, Essential oil, Mouthrinse

## Abstract

**Background:**

The rich diversity of microorganisms in the oral cavity plays an important role in the maintenance of oral health and development of detrimental oral health conditions. Beyond commonly used qualitative microbiome metrics, such as relative proportions or diversity, both the species-level identification and quantification of bacteria are key to understanding clinical disease associations. This study reports the first-time application of an absolute quantitative microbiome analysis using spiked DNA standards and shotgun metagenome sequencing to assess the efficacy and safety of product intervention on dental plaque microbiome.

**Methods:**

In this parallel-group, randomized clinical trial, essential oil mouthrinses, including LISTERINE® Cool Mint Antiseptic (LCM), an alcohol-containing prototype mouthrinse (ACPM), and an alcohol-free prototype mouthrinse (AFPM), were compared against a hydroalcohol control rinse on clinical parameters and the oral microbiome of subjects with moderate gingivitis. To enable a sensitive and clinically meaningful measure of bacterial abundances, species were categorized according to their associations with oral conditions based on published literature and quantified using known amounts of spiked DNA standards.

**Results:**

Multivariate analysis showed that both LCM and ACPM shifted the dysbiotic microbiome composition of subjects with gingivitis to a healthier state after 4 weeks of twice-daily use, resembling the composition of subjects with clinically healthy oral conditions recruited for observational reference comparison at baseline. The essential oil-containing mouthrinses evaluated in this study showed statistically significant reductions in clinical gingivitis and plaque measurements when compared to the hydroalcohol control rinse after 6 weeks of use.

**Conclusions:**

By establishing a novel quantitative method for microbiome analysis, this study sheds light on the mechanisms of LCM mouthrinse efficacy on oral microbial ecology, demonstrating that repeated usage non-selectively resets a gingivitis-like oral microbiome toward that of a healthy oral cavity.

**Trial registration:**

The trial was registered on ClinicalTrials.gov on 10/06/2021. The registration number is NCT04921371.

**Supplementary Information:**

The online version contains supplementary material available at 10.1186/s12903-024-04365-9.

## Background

Advances in DNA sequencing have expanded our understanding of the oral microbiome with its site-specific composition and strong associations with oral health conditions [1–3]. According to the Human Oral Microbiome Database (HOMD), approximately 775 bacterial species reside in the oral cavity, 57% of which are officially named, 13% unnamed but cultivated, and 30% uncultivated phylotypes (eHOMD: http://www.homd.org/). Microbial communities at sites where dental plaque is inaccessible, like interproximal sites, show greater diversity of gingivitis and caries-associated bacteria [4]. Elevated microbial diversity on the tongue is also correlated with an increased representation of malodor-associated bacteria [5]. The salivary microbiome is representative of various oral microenvironments and can be a useful diagnostic indicator of oral health conditions [6] like gingivitis, periodontitis, and caries [7] as well as systemic health conditions [8]. Beyond microbial heterogeneity, the overall abundance of microorganisms plays a critical role in the development and progression of oral diseases. In a healthy mouth, predominant commensal and less abundant pathogenic species exist in a stable ecological equilibrium. Disruption of this balance occurs in the absence or lapse of oral hygiene, leading to dysbiosis, a shift towards an increasingly pathogenic microbial composition [2, 9].

16S rRNA sequencing is a common microbiome profiling approach. Amplification of bacterial 16S ribosomal genes provides high-throughput identification of bacteria via DNA sequence alignment against reference databases. Although this technique detects a wide range of bacterial taxa, it is not able to confidently provide taxonomic identification beyond the genus level [10]. Alternatively, shallow shotgun metagenomic sequencing (SMS) allows for a complete read of the metagenome, consisting of all DNA, both host and microbe, isolated from a sample. With SMS, DNA is randomly broken into smaller fragments, sequenced, and digitally reassembled by aligning overlapping regions. SMS provides more accurate species-level taxonomic and functional profiles of the microbiome than 16S sequencing [11]. However, both methods only generate relative abundance data, delivering difficult to interpret results when comparing sample groups.

Quantifying bacterial cell numbers using DNA-based microbiome profiling can elucidate the complex roles microbiomes play in health and disease. Although various methods have been developed to enable the use of relative abundance data [12], only a few methods can quantify the absolute numbers of bacterial cells [13, 14]. Jian et al. have shown that the product of each sample’s total microbial abundance, determined using quantitative polymerase chain reaction (qPCR), against the relative proportions of each bacteria derived from 16S amplicon sequencing, can provide a simple approximation of bacterial cell numbers [13]. Stammler et al. employed a “spike-in” approach, where controlled amounts of reference DNA from bacteria not normally found in humans were intentionally added in collected specimens prior to sample processing [14]. These “spiked” reference DNAs help to preserve the absolute quantitative microbiological information present in specimens which are otherwise lost due to the multiple DNA amplification events occurring during sample library preparation. Using the linear regression of input spike-in DNA, taxonomic read counts and genome molecular weights, this method enables the caculation of individual bacterial cell numbers.

In the present study, we combined the enhanced species-level identification capability of SMS with spike-in DNA quantification to assess the impact of essential oil (EO)-containing mouthrinses on the oral plaque microbiome. Three EO-containing mouthrinses, including LISTERINE^®^ COOL MINT^®^ (LCM), an alcohol- and EO-containing prototype mouthrinse (ACPM), and an alcohol-free EO prototype mouthrinse (AFPM), were evaluated in subjects with gingivitis for a period of 6 weeks, and then the microbiome of individuals with gingivitis was compared with that of a healthy cohort. To enable measurement of the impacts of oral care products on the clinical plaque microbiome, a literature search was conducted to categorize oral bacteria into commensals, associated with oral health, and pathogens, associated with gingivitis, caries, and malodor. This process of species mapping from the microbiome profiling data with oral health conditions enabled the acquisition of quantitative microbiological results that directly reflect the clinical efficacies of antimicrobial mouthrinses.

## Methods

### Study design

**Fig. 1:**
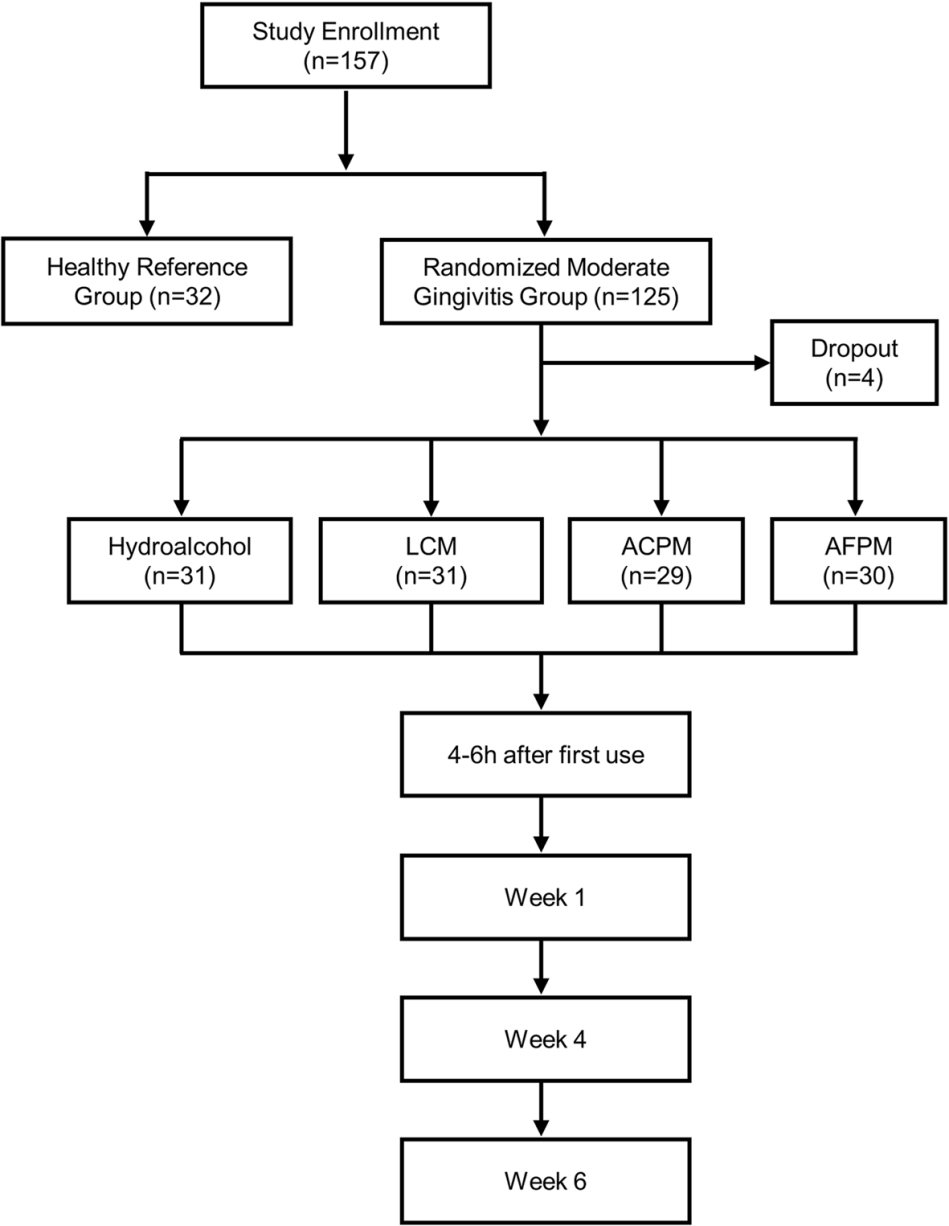
Study design flow chart. *ACPM* alcohol-containing prototype mouthrinse, *AFPM* alcohol-free prototype mouthrinse, *LCM* LISTERINE® COOL MINT. Four patients dropped out of the trial; among them, two withdrew consent, and two were non-compliant

This randomized, controlled, examiner-blind, single-center, parallel-group clinical trial was conducted between October and December 2019 by Salus Research Inc. (Fort Wayne, IN, USA). Subjects in good periodontal health and those with moderate gingivitis were enrolled according to the inclusion/exclusion criteria. At baseline, after abstaining from oral hygiene for at least 8 h, but no more than 18 h, prescreened subjects were given an oral examination of hard and soft tissues and gingivitis and plaque assessments. Periodontally healthy subjects participated only in one baseline assessment. Subjects with moderate gingivitis were randomized to one of four mouthrinses: commercially available US alcohol- and EO-containing LCM as benchmark reference (0.092% eucalyptol, 0.042% menthol, 0.060% methyl salicylate, and 0.064% thymol, 21.6% alcohol) (Johnson & Johnson Consumer Inc., NJ, USA); prototype EO mouthrinses with new sensorial flavor containing alcohol ACPM (0.092% eucalyptol, 0.042% menthol, 0.060% methyl salicylate, and 0.064% thymol, 21.6% alcohol) or without alcohol AFPM (0.092% eucalyptol, 0.042% menthol, 0.060% methyl salicylate, and 0.064% thymol); or a negative control rinse containing 5% hydroalcohol (HA) without any EOs (Fig. 1) [15, 16]. Subjects were given a fluoridated toothpaste (Colgate^®^ Cavity Protection, Colgate-Palmolive Company, NY, USA) and standard soft-bristled toothbrushes. They were instructed to brush twice daily (morning and evening) and to rinse for 30 s with 20 mL of an assigned mouthrinse twice daily for 6 weeks. Clinical assessments, including an oral soft tissue evaluation (OST), the Modified Gingival Index (MGI), Expanded Gingival Bleeding Index (EBI), and six-site Turesky modification of the Quigly-Hein Plaque Index (TPI), were completed at weeks 4 and 6. The primary efficacy endpoints were mean MGI and mean TPI at week 6, and the secondary efficacy endpoints were mean MGI, mean TPI at week 4, and mean EBI and percent bleeding sites at weeks 4 and 6. Supragingival plaque was collected 4–6 h after the first brushing and mouthrinse use at weeks 1, 4, and 6 for exploratory microbiome analysis which was the focus of this publication. All clinical assessments in this trial were performed by the same dental examiners who were trained and calibrated with visual assessment of gingival inflammation, supragingival plaque, and gingival bleeding as measured by MGI, TPI, and EBI.

### Subjects

Healthy adults (≥ 18 years of age) with a minimum of 20 natural teeth, including four molars with scorable facial and lingual surfaces, were included. Specific requirements for the healthy reference subjects included: a whole-mouth mean gingivitis score ≤ 0.75 in the MGI [17], a whole-mouth mean percentage bleeding sites ≤ 3% in the EBI [18], and no teeth with periodontal pocket depths (PPDs) exceeding 3 mm [19–21]. Specific requirements for the randomized subjects with moderate gingivitis included a whole-mouth mean gingivitis score ≥ 1.95 in the MGI, a whole-mouth mean plaque score ≥ 1.95 in the TPI [22, 23], a whole-mouth mean percentage bleeding sites ≥ 10% in the EBI, and ≤ 3 sites with PPDs exceeding 5 mm [24]. Key exclusion criteria were the regular use of chemotherapeutic oral care products; antibiotic, anti-inflammatory, or anticoagulant therapy or professional dental cleaning within 4 weeks prior to baseline; history of significant adverse effects, including allergies after use of oral hygiene products or sensitivity to investigational product ingredients; use of intraoral devices; pregnancy; substance abuse; and participation in any clinical trial within 30 days.

### Clinical assessments

All examinations were conducted in the following order: an OST, the MGI, EBI, plaque collection for microbiome analysis, and TPI. Gingivitis was assessed using the MGI on the buccal and lingual marginal gingiva and interdental papillae of all scorable teeth as follows: 0–normal (absence of inflammation); 1–mild inflammation of any portion of the entire gingival unit; 2–mild inflammation of the entire gingival unit; 3–moderate inflammation of the gingival unit; and 4–severe inflammation of the gingival unit.

Gingival bleeding was assessed according to the EBI, 168 Sites, by inserting a periodontal probe into the gingival crevice and sweeping from distal to mesial around the tooth at an approximate angle of 60°, while in contact with the sulcular epithelium. Each of six gingival areas (distobuccal, midbuccal, mesiobuccal, distolingual, midlingual, and mesiolingual) around each tooth was assessed. After approximately 30 s, bleeding was recorded at each gingival unit according to the following scale: 0–absence of bleeding after 30 s; 1–bleeding after 30 s; and 2–immediate bleeding.

Plaque was assessed by the TPI to include six surfaces (distobuccal, midbuccal, mesiobuccal, distolingual, midlingual, and mesiolingual) of all scorable teeth as follows: 0–no plaque; 1–separate flecks or discontinuous band of plaque around the gingival margin; 2–up to 1 mm continuous band of plaque at the gingival margin; 3–band of plaque wider than 1 mm but less than 1/3 of the surface; 4–plaque covering 1/3 or more, but less than 2/3 of the surface; and 5–plaque covering 2/3 or more of the surface. Safety was assessed via oral examinations at all exam visits to monitor oral soft and hard tissue tolerance to the treatments and via adverse event collection.

### Sample size, treatment allocation, randomization, and blinding

A sample size determination showed that 30 subjects were required per group using a standardized effect size of 0.75 based on previous studies (the difference between treatment population means divided by the population standard deviation) for a two-sided test at the 5% significance level and at 80% power. Assuming a 5% drop-out rate, the trial recruited 32 subjects per group or 128 subjects with moderate gingivitis to ensure trial completion with 120 subjects in the randomized treatment group. Additional 32 subjects in good periodontal health were enrolled, representing the healthy reference group for a baseline assessment only. The randomization schedule for subjects with moderate gingivitis was generated using a validated program created by the Biostatistics Department at Johnson & Johnson Consumer Inc. (Skillman, NJ, USA). Subjects were assigned in an equal allocation to each treatment using a block randomization with a block size of four and were assigned a unique randomization number that sequentially determined the treatment assignment at the baseline visit. To minimize bias, the principal investigator and examiners were blinded to the administered treatments, while the clinical personnel dispensing test products or supervising their use were excluded from subject examinations.

### Plaque sample collection

Supragingival plaque was collected for analysis of the microbiome composition prior to plaque staining with a disclosing dye. Plaque samples were collected from four teeth using a sterile curette by moving supragingivally from the mesiobuccal gingival margin to midbuccal, from distobuccal to midbuccal, and then repeated on the lingual side. For each individual subject, plaque samples were pooled and placed in 250 µL of sterile ultra-pure grade phosphate-buffered saline, pH 7.2, and then stored at − 80 °C. The preferentially sampled teeth were the upper right first molar, upper right lateral incisor, lower left second molar, and lower left lateral incisor, which also met the inclusion and exclusion criteria for periodontally healthy subjects versus subjects with moderate gingivitis. Adjacent teeth that met the selection criteria were used as substitutes if the teeth were missing.

### Shotgun metagenomic sequencing

DNA isolation, library preparation, and sequencing were carried out at CosmosID (Germantown, MD) using a vendor-optimized protocol. Briefly, plaque samples were spiked with *Truepera radiovictrix, Imtechella halotolerans*, and *Allobacillus halotolerans* using ZymoBIOMICS Spike-in Control II (Zymo Research, Irvine, CA) to enable bacterial cell number quantification. To enhance bacterial cell lysis, plaque samples were incubated with MetaPolyzyme at 35 °C for 12 h, and DNA was extracted employing the ZymoBIOMICS DNA MicroPrep with bead-beating according to the manufacturer’s instructions. The concentration of all DNA samples and libraries were determined using the Qubit dsDNA HS assay and Qubit 4 fluorometer (ThermoFisher Scientific, Waltham, MA). To construct DNA libraries, 1 ng of input genomic DNA was fragmented, amplified, and indexed utilizing Nextera XT DNA Library Preparation and Nextera Indexing Kit (Illumina, San Diego, CA). DNA libraries were purified using AMPure magnetic beads (Beckman Coulter, Brea, CA) and then normalized for equimolar pooling. Sequencing was performed with a HiSeq sequencer (Illumina), targeting a coverage of 3 − 4 million paired-end 2 × 150 bp reads.

### Statistical analysis of clinical results

Subject demographic and baseline characteristics, including clinical assessment scores (MGI, TPI, and EBI), were compared across treatment groups using analysis of variance (ANOVA), Chi-square test, or Fisher’s exact test. Efficacy analysis was based on the full analysis set following the Intent-to-Treat principle. No imputation of missing data was performed. Statistical comparisons for primary and secondary clinical efficacy endpoints were carried out using a repeated measures mixed model, including terms for treatment and visit and the corresponding baseline value as a covariate.

### Computational microbiome analysis

To enable bacterial cell number quantification, plaque samples were spiked with ZymoBIOMICS Spike-in Control II (Zymo Research, Irvine, CA). Raw sequence reads were processed, quality filtered and taxonomically assigned by CosmosID Inc (Germantown, MD). Bacterial diversity analyses were performed using R-3.6.1 [25]. Alpha-diversity metrics were assessed utilizing *vegan* 2.5.6 [26] and included observed richness and Shannon-Weaver diversity indices at the species taxonomic level. Statistical significance was tested using mixed model repeated measures with a baseline covariate and terms for treatment, visit, treatment-by-visit and baseline-by-visit, and unstructured within-subject covariance.

Beta-diversity analysis was performed using *phyloseq* 1.28.0 [27] to compute the phylogenetic distance matrix by the weighted UniFrac [28] for ordination utilizing principal coordinate analysis. The input phylogenetic tree was constructed using GenBank based on the data taxonomy table. Significance testing of factors and interactions affecting bacterial compositions was performed employing PERMANOVA [29] in *adonis* in *vegan* [26].

For quantification of bacterial abundances, standard calibration curves of spike-in bacteria were evaluated for each sample based on the input amount of control DNA against their output relative abundance values. The amount of DNA for each taxon was computed using linear regression of spike-in control DNA for each sample, and bacterial abundances were computed employing the genome molecular weights specific for each taxon from GenBank. The resulting bacterial abundances were expressed in units of calculated microbial units (CMUs) and were represented in log10 where appropriate. For differential abundance testing of individual bacterial species, these log10 abundance values were used in two-sample Wilcoxon rank sum tests comparing healthy vs. gingivitis subjects at baseline or comparing each mouthrinse against the hydroalcohol negative control for week 4 and week 6 visits.

To assess the product impact, bacterial taxa were classified into specific categories based on their association with health conditions: commensals, malodor associated, gingival disease associated, acidogenic, and systemic pathogens. The bacterial classification was assigned based on scientific literature review and annotations from the HOMD [30]. The abundances of bacterial species associated with these different categories were log10-transformed and pooled per sample, and the means were reported for group comparisons.

## Results

### Subjects

Of the 157 subjects enrolled in this clinical trial, four subjects discontinued (Fig. 1). Those who completed the trial included 32 subjects in good periodontal health (healthy) and 121 subjects with moderate gingivitis, randomized into four treatment arms: 31 in the LCM group, 29 in the ACPM group, 30 in the alcohol-free EO-containing AFPM group, and 31 in the negative control 5% HA rinse group. The mean (SD) age of the subjects was 43.2 (13.3) years, and the majority were females (74.5%), Caucasian (89.5%), and non-smokers (100%). The overall clinical parameters for healthy subjects at baseline with regards to the mean (SD) MGI, TPI, and EBI were 0.438 (0.115), 2.530 (0.392), and 0.018 (0.013), respectively, whereas, for subjects with moderate gingivitis, these scores were 2.530 (0.255), 3.001 (0.400), and 0.356 (0.191), respectively (Table 1).

**Table 1:** Subject demographic and baseline characteristics

Parameters	Healthy (*n* = 32)	HA (*n* = 31)	LCM (*n* = 31)	ACPM (*n* = 29)	AFPM (*n* = 30)	Overall *p*-value
Mean age (range), years	45.9 (25 − 66)	41.9 (20 − 64)	42.1 (22 − 72)	41.1 (20 − 69)	44.5 (18 − 70)	0.591^a^
Sex, n (%)						
Male	8 (25.0)	8 (25.8)	7 (22.6)	8 (27.6)	8 (26.7)	0.994^b^
Female	24 (75.0)	23 (74.2)	24 (77.4)	21 (72.4)	22 (73.3)	
Body mass index (range)	26.8 (17.3 − 40.9)	30.1 (20.4 − 64.1)	30.8 (21.0 − 42.0)	31.4 (20.1 − 55.7)	29.7 (21.9 − 39.9)	0.101^a^
Race, n (%)						
White	27 (84.4)	29 (93.5)	29 (93.5)	26 (89.7)	26 (86.7)	0.724^c^
Black or African American	5 (15.6)	2 (6.5)	2 (6.5)	3 (10.3)	4 (13.3)	
Smoker, n (%)						
No	32 (100.0)	31 (100.0)	31 (100.0)	29 (100.0)	30 (100.0)	0.997^b^
Whole-mouth scores ± SD						
Baseline mean MGI	0.438 ± 0.115	2.510 ± 0.289	2.614 ± 0.242	2.578 ± 0.221	2.416 ± 0.223	< 0.001^a^
Baseline mean TPI	2.530 ± 0.392	2.984 ± 0.434	3.111 ± 0.356	3.009 ± 0.380	2.898 ± 0.417	< 0.001^a^
Baseline mean EBI	0.018 ± 0.013	0.339 ± 0.171	0.378 ± 0.192	0.356 ± 0.153	0.348 ± 0.241	< 0.001^a^

^a^*p*-values are based on an ANOVA model with term for treatment

^b^*p*-values are based on Chi-square test

^c^*p*-values are based on Fisher’s exact test

*ACPM* alcohol-containing prototype mouthrinse, *AFPM* alcohol-free prototype mouthrinse, *HA* hydroalcohol negative control, *LCM* LISTERINE COOL MINT Antiseptic mouthrinse

### Clinical efficacy and safety

After twice daily use for 6 weeks, subjects with moderate gingivitis showed significant improvements in clinical signs of gingivitis, plaque, and bleeding in all three treatment groups (Table 2). Assessment of gingival inflammation by whole-mouth mean MGI demonstrated that all mouthrinses reduced gingivitis by at least 37.4% compared with HA. Although ACPM demonstrated significant reductions only in MGI after 6 weeks, LCM and AFPM showed significant reductions in MGI earlier, after 4 weeks (Table 2). Additionally, LCM and ACPM demonstrated significant reductions in dental plaque by at least 22% after 4 weeks, with further reductions after 6 weeks (Table 2). AFPM established a significant 14% plaque reduction after 6 weeks (Table 2). Consistent with reductions in gingivitis and plaque, all mouthrinses resulted in a bleeding reduction of at least 36.3% compared with the HA negative control (Table 2). All treatments in this trial were well tolerated.

**Table 2:** Clinical efficacy: differences in the adjusted whole-mouth mean (SE) at each visit

Parameters	HA	LCM	ACPM	AFPM
**Modified Gingival Index**				
Baseline	2.511 (0.050)	2.614 (0.044)	2.578 (0.041)	2.417 (0.041)
Week 4	1.888 (0.075)	1.424 (0.076)	1.758 (0.078)	1.652 (0.078)
Week 6	2.065 (0.087)	1.148 (0.089)	1.294 (0.091)	1.226 (0.091)
% Difference vs. HA (*p*-value)				
Week 4		-24.6% (< 0.001^a^)	-6.9% (0.233^a^)	-12.5% (0.030^a^)
Week 6		-44.4% (< 0.001^a^)	-37.4% (< 0.001^a^)	-40.6% (< 0.001^a^)
**Plaque Index**				
Baseline	2.983 (0.076)	3.111 (0.064)	3.009 (0.071)	2.897 (0.076)
Week 4	2.638 (0.078)	1.986 (0.078)	2.040 (0.080)	2.482 (0.080)
Week 6	2.988 (0.069)	2.205 (0.070)	2.174 (0.071)	2.570 (0.071)
% Difference vs. HA (*p*-value)				
Week 4		-24.7% (< 0.001^a^)	-22.6% (< 0.001^a^)	-5.9% (0.164^a^)
Week 6		-26.2% (< 0.001^a^)	-27.3% (< 0.001^a^)	-14.0% (< 0.001^a^)
**Expanded Bleeding Index**				
Baseline	0.340 (0.032)	0.378 (0.035)	0.355 (0.028)	0.347 (0.044)
Week 4	0.285 (0.028)	0.245 (0.028)	0.266 (0.029)	0.266 (0.028)
Week 6	0.360 (0.022)	0.223 (0.022)	0.229 (0.022)	0.216 (0.022)
% Difference vs. HA (*p*-value)				
Week 4		-14.1% (0.307)	-6.8% (0.628)	-6.8% (0.622)
Week 6		-38.0% (< 0.001^a^)	-36.3% (< 0.001)	-40.0% (< 0.001)

^a^*p*-values are based on a repeated measures mixed model, including terms for treatment and visit, the corresponding baseline value as a covariate, and treatment-by-visit and baseline-by-visit terms

*ACPM* alcohol-containing prototype mouthrinse, *AFPM* alcohol-free prototype mouthrinse, *HA* hydroalcohol negative control, *LCM* LISTERINE COOL MINT Antiseptic mouthrinse

### Microbial profiling and mapping to oral health conditions

From the SMS data, 856 unique taxa were identified at the species level. These taxa were classified according to their clinical relevance via extensive clinical and scientific literature review coupled with HOMD data (Table 3 and Additional File 1). Among them, 386 were unclassified, 257 were identified as extraoral bacterial species, and 213 were identified as human oral bacterial species. To enable quantitative microbiome assessment after mouthrinse use, these 213 bacterial species were subdivided according to their oral health-associated outcome, including commensals and those associated with gingivitis, periodontitis, malodor, and caries. There was some species overlap across these different categories (Additional File 1). Therefore, quantification of bacteria associated with specific oral health conditions was confined to each individual category to avoid artificial value inflation.

**Table 3:** Microbial profiling and classification summary

Number of samples analyzed	643
**Number of taxa identified**	856
Unknown or unclassified species	386
Contaminants	257
Oral bacterial species	213
**Clinically relevant classifications**	
Commensals	138
Gingivitis	32
Periodontitis	55
Malodor	29
Caries	23
Oral health-associated	9
Opportunistic pathogens	85
Infectious pathogens	23
**Number of species for validation**	
Total anaerobe count (Schaedler blood or ETSA equivalent)	153
Fusobacteria count (CVE equivalent)	3
Streptococci count (MS equivalent)	22
* Actinomyces* (CFAT equivalent)	2

*CFAT* cadmium sulfate fluoride acridine trypticase, *CVE* crystal-violet erythromycin, *ETSA* enriched trypticase soy agar, *MS* mitis-salivarius

### Short-term and long-term impacts on supragingival plaque microbiome

The accuracy of bacterial cell numbers calculated with the spike-in control approach was evaluated against colony-counting and 16S rRNA qPCR (Table 4). The sum of oral bacterial species (Table 3) representing the different selectivity of growth media showed similar abundance results to reported colony-counting values (Table 4). Bacterial counts reported for 16S qPCR were generally one order of magnitude higher than those for the spike-in approach or colony-counting, likely due to the non-specific detection of total bacterial species including non-culturable, aerobic, transient extra-oral species and non-specific amplicons.

**Table 4:** Microbial load at baseline compared with culture-based colony counting and 16S rRNA qPCR. Abundance values, adjusted for whole teeth and log_10_ transformed, are presented

Selective Media Type	This Study (SMS)	Reported Colony Counting	Reported 16 S rRNA qPCR	References
**Healthy Subjects**				
Total anaerobes	8.789	8.280–8.497	9.903	[31–33]
* Fusobacteria*	4.540	5.674–5.687	7.703	[31–33]
* Streptococci*	7.944	8.342–8.352	-	[33]
* Actinomyces*	8.216	6.371–6.390	-	[33]
**Gingivitis Subjects**				
Total anaerobes	8.856	8.423–8.923	10.003–10.203	[31, 34–36]
* Fusobacteria*	5.539	6.313–7.459	8.203	[31, 34–36]
* Streptococci*	8.015	7.890–8.010	-	[35]
* Actinomyces*	8.282	8.253–8.376	-	[35]

*qPCR* quantitative polymerase chain reaction

**Fig. 2:**
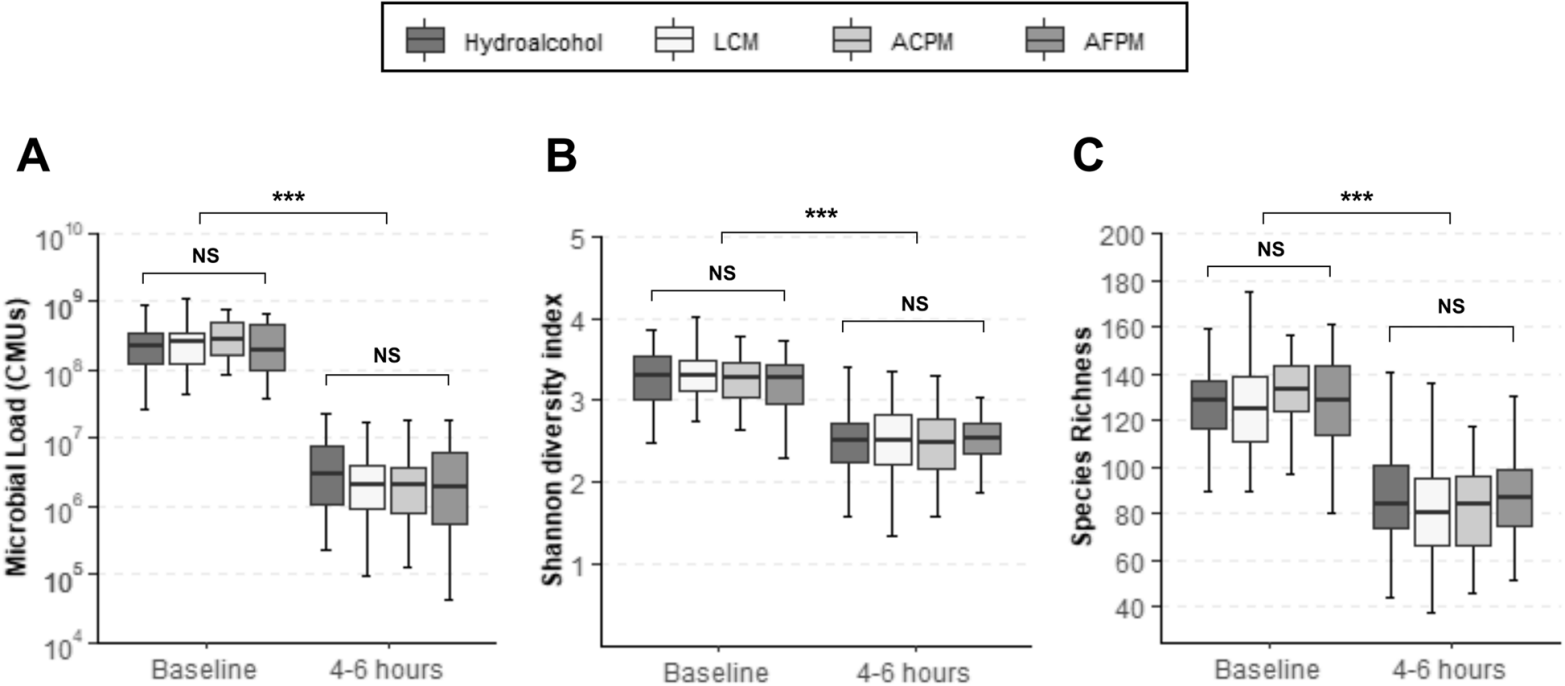
Changes in the total microbial load and diversity of supragingival plaque microbiome in moderate gingivitis 4–6 h after dental prophylaxis and rinsing with a mouthrinse for 30 s. Box plots of the total microbial load **A**, Shannon-Weaver diversity index **B**, and observed species richness **C** are shown. Error bars represent the range of values. ***p < 0.001. *ACPM* alcohol-containing prototype mouthrinse, *AFPM* alcohol-free prototype mouthrinse, *LCM* LISTERINE® COOL MINT, *NS* non-significant

**Fig. 3:**
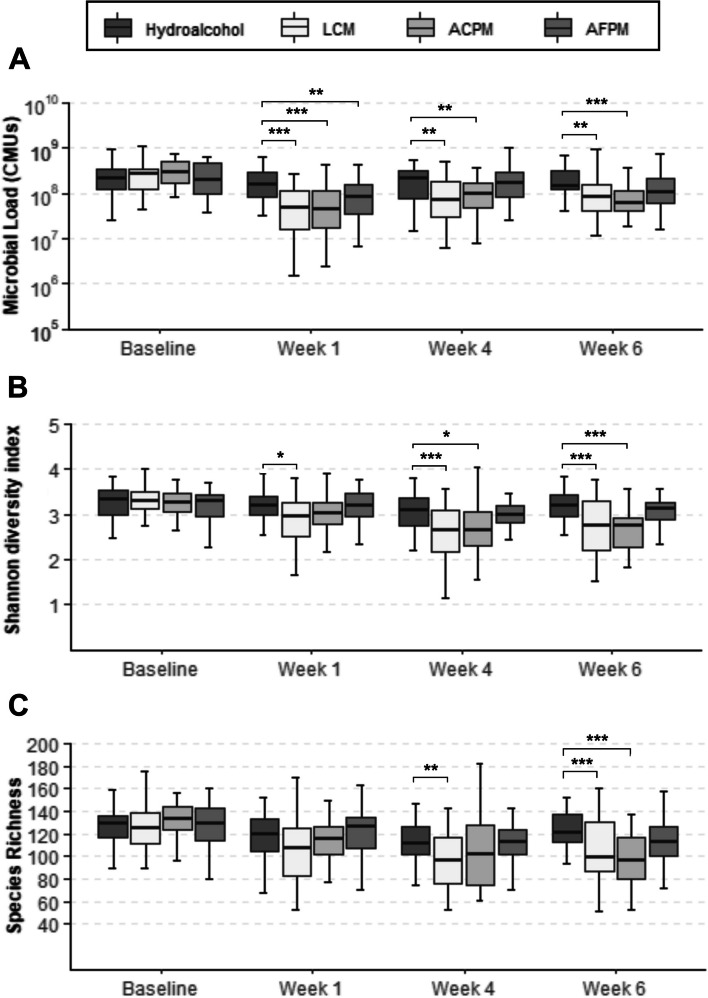
Time-resolved changes in the microbial load and diversity of supragingival plaque in subjects with moderate gingivitis after twice daily brushing followed by rinsing with a mouthrinse for 30 s. Box plots of the total microbial load **A**, Shannon-Weaver diversity index **B**, and observed species richness **C** are shown. Error bars represent the range of values. *p < 0.05, **p < 0.01, ***p < 0.001 (mixed model repeated measures with baseline covariate and terms for treatment, visit, treatment-by-visit and baseline-by-visit, and unstructured within-subject covariance). *ACPM* alcohol-containing prototype mouthrinse, *AFPM* alcohol-free prototype mouthrinse, *LCM* Listerine® COOL MINT

**Fig. 4:**
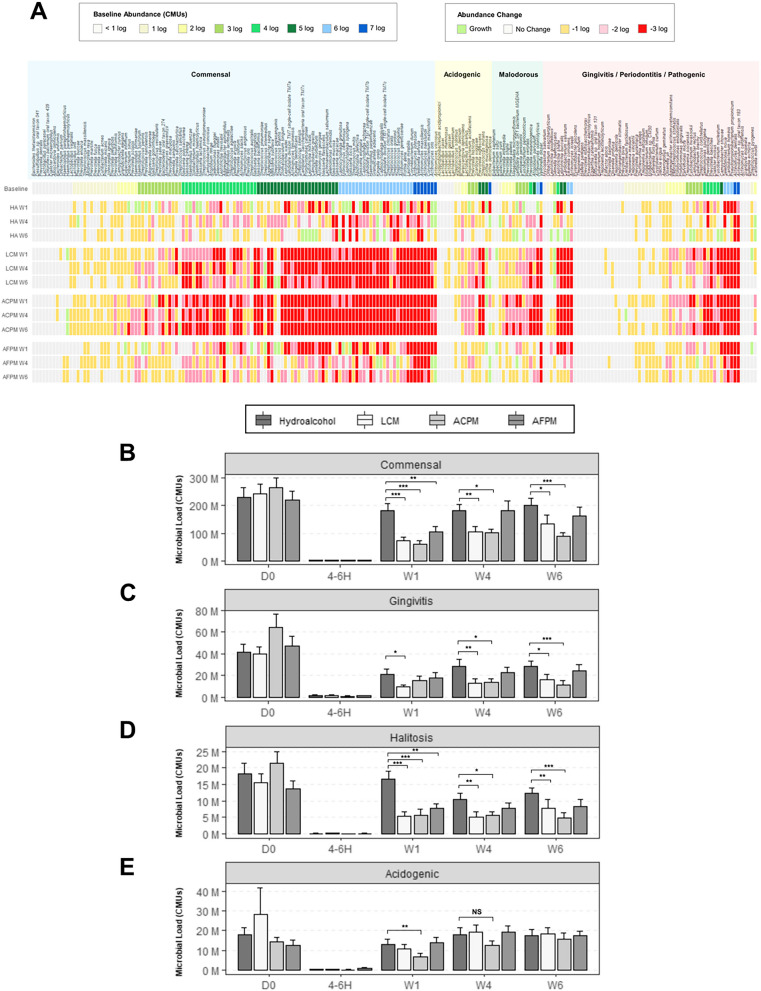
Impact of mouthrinse use on different classifications of bacteria in the supragingival plaque of subjects with moderate gingivitis. **A** Heatmap showing changes in the abundance of individual bacterial species in supragingival plaque of subjects with moderate gingivitis at different time points compared to baseline. Baseline abundance values represent the initial number of bacterial cells at the start of the study expressed as means of log_10_ CMUs. Changes in bacterial cell numbers are expressed as means of paired differences in log_10_ CMUs at each time point compared to baseline. **B** Abundance values represent means of total CMUs from bacterial species that are oral commensals or are **C** associated with gingivitis, **D** volatile-sulfur compound − producing, **E** or acidogenic. Subjects with active caries or grossly carious lesions were not included in this trial. Error bars denote the standard error of the mean. *ACPM* alcohol-containing prototype mouthrinse, *AFPM* alcohol-free prototype mouthrinse, *LCM* LISTERINE COOL MINT Antiseptic mouthrinse, *D0* Day 0/baseline, *W* week, *4–6 H* 4–6 h after dental prophylaxis and mouthrinse use; *W* week. *p < 0.05, **p < 0.01, ***p < 0.001 (mixed model repeated measures with baseline covariate and terms for treatment, visit, treatment-by-visit, and baseline-by-visit)

The subjects who participated in this trial maintained reduced levels of plaque bacteria 4–6 h after the initial prophylaxis and first 30 s rinse (Fig. 2A-C). The reductions observed for the interventions were not significantly different from the known bacterial reduction effects of dental prophylaxis represented by the HA control. A longitudinal assessment of twice daily mouthrinse use on the supragingival plaque microbiota was also performed. Subjects with moderate gingivitis in the LCM and ACPM groups experienced significant reductions in total microbial load (TML) after 1, 4, and 6 weeks (Fig. 3A). The AFPM group only demonstrated a significant TML reduction after 1 week. The HA negative control group had no significant changes in TML compared to baseline (Fig. 3A). Analysis of alpha diversity showed the supragingival plaque of subjects in the LCM and ACPM groups had significant reductions in the Shannon-Weaver diversity index after 4 and 6 weeks of mouthrinse use (Fig. 3B). However, AFPM maintained similar microbial diversity as the HA negative control group. Analysis of the observed richness index showed significant decreases in the number of bacterial species in the LCM and ACPM groups after 6 weeks of mouthrinse use (Fig. 3C). To better understand the changes occurring in the microbiome composition, the abundance of individual bacterial species at each visit was compared with that at baseline. Log_10_ CMU results showed a preponderance of commensal species compared with those associated with clinical signs of gingivitis (Fig. 4A Baseline Commensal vs. Gingivitis/Periodontitis Species). Furthermore, the TML reduction patterns for subjects assigned to EO mouthrinses were indiscriminate across all bacterial categories. Considering the polymicrobial etiology of oral health issues, the impact of mouthrinse use was also assessed by comparing the sums of bacterial abundances by clinical relevance group (Fig. 4B-E; Table 5). Commensal, gingivitis and halitosis associated species were significantly reduced by twice daily use of LCM or ACPM compared with the HA group after 1, 4, and 6 weeks (Fig. 4B-E) The AFPM group showed no statistically significant reductions. Furthermore, no significant differences were observed for acidogenic species between the mouthrinse treatments and the HA negative control group, reflecting the clinical trial eligibility criteria that excluded subjects at risk of caries development.

**Table 5:** List of bacterial species significantly impacted by mouthrinse use (*p* < 0.05) after 4 and 6 weeks. The presented abundance values are means of log_10_ CMUs. Significance was calculated using unpaired two-sample Wilcoxon rank sum test. Species that are not significantly affected by any of the treatments are not shown

Species	Association	Healthy	HA Week 4	LCM Week 4	ACPM Week 4	AFPM Week 4	HA Week 6	LCM Week 6	ACPM Week 6	AFPM Week 6
*Aggregatibacter actinomycetemcomitans*	Gingivitis	1.020	1.074	0.000	-	0.253	1.690	0.262	-	0.490
*Campylobacter rectus*	Gingivitis	2.755	2.847	1.748	-	-	3.436	2.407	-	-
*Cardiobacterium hominis*	Gingivitis	5.909	5.651	5.180	5.196	-	5.959	5.272	5.116	-
*Prevotella loescheii*	Gingivitis	2.664	3.560	2.158	-	-	3.985	2.553	-	-
*Pseudopropionibacterium propionicum*	Gingivitis	5.474	6.009	5.215	5.310	-	6.068	5.455	5.258	-
*Selenomonas noxia*	Gingivitis	5.596	5.224	4.003	-	-	5.709	4.752	-	-
*Actinomyces israelii*	Gingivitis	5.317	5.629	4.825	4.877	-	5.602	4.862	4.887	-
*Cardiobacterium valvarum*	Gingivitis	4.279	4.572	2.901	-	-	4.877	3.986	-	-
*Eikenella corrodens*	Gingivitis	5.252	5.289	4.529	4.453	-	5.319	4.655	4.176	-
*Leptotrichia buccalis*	Gingivitis	3.365	3.316	1.164	-	-	3.895	2.197	-	-
*Leptotrichia shahii*	Gingivitis	2.886	2.942	0.763	1.799	-	3.616	1.812	1.587	-
*Actinomyces odontolyticus*	Malodor	5.350	5.622	4.927	5.004	-	5.546	4.777	4.660	-
*Prevotella melaninogenica*	Malodor	3.371	3.789	2.517	-	-	4.149	2.608	-	-
*Veillonella dispar*	Malodor	6.559	6.377	5.811	6.008	-	6.654	5.820	5.795	-
*Abiotrophia defectiva*	Commensal	5.207	4.261	2.804	-	-	4.503	3.428	-	-
*Actinomyces johnsonii*	Commensal	6.191	6.344	5.722	5.875	-	6.414	5.784	5.754	-
*Actinomyces massiliensis*	Commensal	6.502	6.405	5.322	5.629	-	6.447	5.567	5.605	-
*Actinomyces naeslundii*	Commensal	6.650	6.893	6.201	6.229	-	6.897	6.204	6.238	-
*Actinomyces slackii*	Commensal	4.109	4.287	3.585	-	-	4.277	3.740	-	-
*Actinomyces timonensis*	Commensal	4.815	4.813	4.441	4.434	-	4.819	4.333	4.343	-
*Aggregatibacter aphrophilus*	Commensal	3.601	3.268	1.903	2.197	-	3.965	2.378	1.795	-
*Aggregatibacter segnis*	Commensal	3.991	3.323	1.621	-	-	4.128	2.469	-	-
*Campylobacter gracilis*	Commensal	4.653	4.811	3.831	-	-	4.975	4.483	-	-
*Candidate division TM7a*	Commensal	4.268	4.067	1.909	2.544	-	4.360	2.972	2.476	-
*Capnocytophaga gingivalis*	Commensal	5.863	5.910	5.143	5.311	-	5.994	5.415	4.908	-
*Capnocytophaga granulosa*	Commensal	5.289	5.401	3.721	4.143	-	5.479	4.334	3.944	-
*Capnocytophaga ochracea*	Commensal	5.479	5.363	4.251	4.231	-	5.588	4.709	4.456	-
*Capnocytophaga sputigena*	Commensal	5.347	5.545	4.642	4.744	-	5.485	4.856	4.428	-
*Corynebacterium durum*	Commensal	6.096	6.224	5.367	5.272	-	6.280	5.487	5.488	-
*Corynebacterium matruchotii*	Commensal	6.824	6.591	5.441	5.839	-	6.831	5.927	5.870	-
*Haemophilus haemolyticus*	Commensal	3.878	3.442	2.140	2.363	2.058	4.190	2.131	2.309	2.513
*Haemophilus influenzae*	Commensal	3.430	3.119	2.040	1.490	1.938	3.521	1.861	1.721	2.413
*Haemophilus parainfluenzae*	Commensal	5.934	5.730	4.695	4.773	5.246	6.091	4.949	4.760	5.210
*Haemophilus pittmaniae*	Commensal	2.835	2.428	1.030	0.470	1.153	2.911	1.018	0.274	0.986
*Haemophilus sputorum*	Commensal	2.454	2.071	1.215	0.652	-	3.023	1.269	0.270	-
*Kingella denitrificans*	Commensal	4.505	4.336	3.089	3.124	-	4.532	3.682	3.077	-
*Kingella oralis*	Commensal	6.133	5.755	5.197	5.281	-	5.870	5.275	5.266	-
*Lachnoanaerobaculum saburreum*	Commensal	4.910	4.671	3.289	3.545	-	5.201	3.920	3.561	-
*Lautropia mirabilis*	Commensal	6.143	6.163	4.937	5.150	-	6.217	5.075	4.778	-
*Leptotrichia hofstadii*	Commensal	4.737	4.776	1.663	3.012	3.874	5.031	3.084	2.486	3.682
*Leptotrichia trevisanii*	Commensal	2.998	3.133	1.003	1.975	-	3.749	2.044	1.517	-
*Leptotrichia wadei*	Commensal	4.246	4.202	1.499	2.120	-	4.702	2.507	2.184	-
*Neisseria bacilliformis*	Commensal	4.441	3.620	2.665	-	-	4.326	3.157	-	-
*Neisseria cinerea*	Commensal	2.526	2.558	1.398	-	-	3.249	1.734	-	-
*Neisseria elongata*	Commensal	5.281	5.470	4.450	4.193	-	5.727	4.941	4.265	-
*Neisseria flavescens*	Commensal	4.522	4.171	2.738	2.798	-	4.502	2.800	2.480	-
*Neisseria lactamica*	Commensal	2.809	2.848	1.819	-	-	3.674	2.313	-	-
*Neisseria macacae*	Commensal	4.631	4.771	3.746	3.307	-	4.858	4.129	3.357	-
*Neisseria meningitidis*	Commensal	3.800	4.397	2.736	2.617	-	4.437	3.241	2.524	-
*Neisseria polysaccharea*	Commensal	2.502	2.539	1.520	-	-	3.218	1.727	-	-
*Neisseria subflava*	Commensal	4.108	3.783	2.270	2.403	-	4.451	2.798	2.375	-
*Porphyromonas catoniae*	Commensal	4.908	4.625	3.322	-	-	5.035	3.976	-	-
*Prevotella salivae*	Commensal	1.890	2.155	0.961	-	-	2.954	1.382	-	-
*Rothia aeria*	Commensal	6.148	6.328	6.062	5.995	-	6.426	6.121	5.928	-
*Selenomonas artemidis*	Commensal	4.870	4.829	3.628	3.398	-	5.158	3.649	3.653	-
*Selenomonas flueggei*	Commensal	3.142	2.901	1.192	-	-	3.921	2.178	-	-
*Streptococcus cristatus*	Commensal	6.027	6.041	4.858	5.179	5.587	6.113	5.187	5.128	5.669
*Streptococcus mitis*	Commensal	5.509	5.453	5.121	5.130	-	5.744	5.199	5.039	-
*Streptococcus sinensis*	Commensal	4.776	4.728	3.426	4.025	4.230	4.784	4.078	4.167	4.202
*Veillonella atypica*	Commensal	4.049	3.480	2.125	-	-	4.375	2.591	-	-
*Veillonella parvula*	Commensal	6.639	6.439	5.885	6.102	-	6.711	5.915	5.895	-
*Neisseria gonorrhoeae*	Commensal	1.904	2.088	-	0.856	-	2.596	-	1.276	-
*Streptococcus pneumoniae*	Commensal	4.524	4.546	-	4.023	-	4.537	-	4.004	-

### Return from dysbiosis to healthier levels

**Fig. 5:**
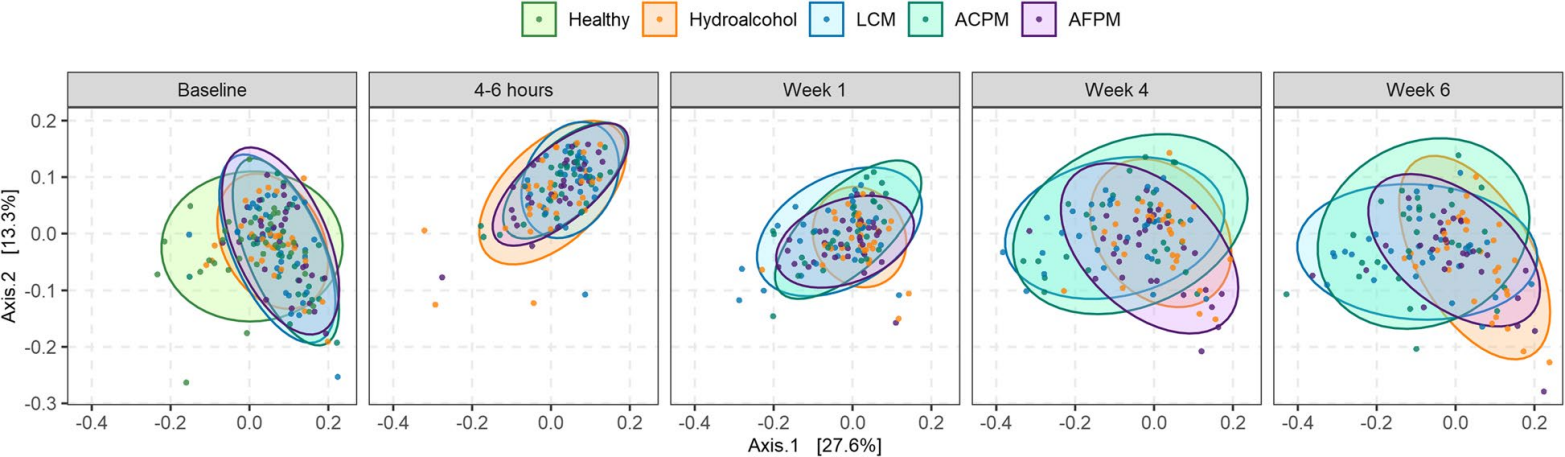
Time-resolved changes in the beta diversity of supragingival plaque microbiome comparing subjects with moderate gingivitis randomized to different mouthrinse treatment groups against reference cohorts with naturally healthy oral cavities at baseline. Sample ordination was performed with the weighted UniFrac distance computed at the species taxonomic level, using CMUs for bacterial abundance, and graphed in a two-dimensional principal coordinate plot. *ACPM* alcohol-containing prototype mouthrinse, *AFPM* alcohol-free prototype mouthrinse, *LCM* LISTERINE COOL MINT Antiseptic mouthrinse

The longitudinal impacts on the supragingival plaque microbiome of subjects with moderate gingivitis were compared against the naturally healthy reference cohort at baseline. Analysis of beta diversity showed that the microbiota of subjects with gingivitis were distinct from those of the healthy reference at baseline (Fig. 5). Differential abundance testing revealed that several species were significantly more abundant in subjects with gingivitis, including commensals and key pathogens associated with gingivitis, such as *Fusobacterium nucleatum, A. actinomycetemcomitans*, and *Prevotella* species (Table 6). Comparisons at post-baseline visits demonstrated changes in the microbiome composition of subjects with gingivitis in the LCM and ACPM groups, resembling the healthy cohorts at baseline, whereas the microbiome composition of the AFPM and HA groups resembled that of subjects with gingivitis at baseline (Fig. 5). Further examination of individual bacteria abundances showed that many oral bacterial species were significantly reduced to levels comparable or lower than those found in healthy cohorts (Table 5).

**Table 6:** List of bacterial species with significant differences in bacterial abundance between subjects with moderate gingivitis and naturally healthy reference cohorts at baseline. The presented abundance values are means of log_10_ CMUs, whereas the *p-*values represent unpaired two-sample Wilcoxon rank sum test

Species	Healthy Subjects	Subjects with Gingivitis	Difference	*p*-values
*Abiotrophia defectiva*	5.207	5.756	0.549	0.039
*Actinobaculum sp. oral taxon 183*	6.228	6.783	0.555	0.030
*Actinomyces dentalis*	5.828	6.518	0.690	0.001
*Actinomyces georgiae*	4.266	5.201	0.935	0.023
*Actinomyces gerencseriae*	5.896	6.345	0.449	0.007
*Actinomyces israelii*	5.317	5.758	0.441	0.015
*Actinomyces johnsonii*	6.191	6.561	0.369	0.004
*Actinomyces naeslundii*	6.650	6.952	0.302	0.028
*Aggregatibacter actinomycetemcomitans*	1.020	1.951	0.931	0.032
*Aggregatibacter segnis*	3.991	4.669	0.679	0.022
*Atopobium rimae*	2.823	3.873	1.050	0.004
*Bacteroidetes oral taxon 274*	1.818	2.927	1.109	0.028
*Campylobacter showae*	3.780	4.522	0.742	0.009
*Candidate division TM7 single-cell isolate TM7a*	4.268	4.889	0.621	0.036
*Candidate division TM7 single-cell isolate TM7b*	4.892	5.879	0.987	0.032
*Candidate division TM7 single-cell isolate TM7c*	5.384	6.089	0.706	0.033
*Candidatus Saccharibacteria oral taxon TM7x*	3.929	5.055	1.126	0.002
*Cardiobacterium hominis*	5.909	6.273	0.365	0.041
*Cutibacterium acnes*	0.126	1.099	0.972	0.003
*Dialister invisus*	3.096	4.251	1.155	0.007
*Eikenella corrodens*	5.252	5.579	0.328	0.046
*Eubacterium brachy*	2.177	3.384	1.207	0.031
*Fusobacterium nucleatum*	2.968	4.472	1.504	0.001
*Gemella morbillorum*	4.382	5.155	0.773	0.012
*Kingella denitrificans*	4.505	4.982	0.476	0.003
*Leptotrichia buccalis*	3.365	4.515	1.149	0.002
*Leptotrichia goodfellowii*	0.674	1.619	0.945	0.022
*Leptotrichia shahii*	2.886	3.735	0.849	0.030
*Leptotrichia trevisanii*	2.998	3.996	0.998	0.035
*Morococcus cerebrosus*	4.779	5.538	0.759	0.009
*Neisseria cinerea*	2.526	3.635	1.109	0.004
*Neisseria flavescens*	4.522	5.203	0.681	0.008
*Neisseria lactamica*	2.809	3.752	0.943	0.004
*Neisseria macacae*	4.631	5.448	0.817	0.011
*Neisseria meningitidis*	3.800	4.498	0.698	0.024
*Neisseria mucosa*	4.364	5.284	0.920	0.001
*Neisseria polysaccharea*	2.502	3.515	1.013	0.008
*Neisseria sicca*	4.811	5.692	0.881	0.005
*Neisseria subflava*	4.108	4.672	0.563	0.032
*Prevotella intermedia*	1.384	2.147	0.763	0.045
*Prevotella loescheii*	2.664	4.186	1.522	0.002
*Prevotella maculosa*	2.470	3.351	0.880	0.047
*Prevotella melaninogenica*	3.371	4.214	0.844	0.029
*Prevotella nigrescens*	3.976	4.514	0.538	0.011
*Prevotella oris*	4.004	4.443	0.439	0.025
*Prevotella oulorum*	3.067	4.332	1.266	0.008
*Prevotella pallens*	1.265	2.196	0.931	0.031
*Prevotella saccharolytica*	2.149	3.397	1.247	0.010
*Prevotella salivae*	1.891	2.894	1.003	0.013
*Prevotella scopos*	1.248	2.097	0.849	0.030
*Prevotella veroralis*	1.876	3.339	1.463	0.002
*Pseudopropionibacterium propionicum*	5.474	6.282	0.809	0.000
*Rothia dentocariosa*	7.084	6.858	-0.226	0.004
*Selenomonas sputigena*	2.800	3.579	0.779	0.025
*Solobacterium moorei*	2.235	3.678	1.443	0.002
*Streptococcus agalactiae*	3.553	4.243	0.690	0.030
*Treponema socranskii*	1.671	2.763	1.092	0.023

## Discussion

The unique formulation of Listerine mouthrinses with a fixed combination of EOs (menthol, thymol, eucalyptol, and methyl salicylate) provides a broad-spectrum bactericidal action that is demonstrated to be safe and efficacious over decades of published clinical studies [15, 16, 37]. In this study, we aimed to characterize the effects of EO-containing mouthrinses on the microbiome of oral plaque using a novel method of quantitative analysis that combined the enhanced species-level identification capability of SMS with spike-in DNA quantification. The data from this study provides a sensitive and detailed assessment of the oral microbiome not previously available in studies of oral care products [38, 39]. This first-time application of quantitative microbiome analysis describes how the broad-spectrum antimicrobial effects of EOs skew plaque regrowth towards an early colonizing state, and shift the microbiome towards health with repeated use.

All three EO-containing mouthrinses resulted in significant reductions in clinical plaque and gingivitis measurements when compared to the HA control over 6 weeks of use consistent with previously published clinical studies (Table 2) [15, 16, 37]. The prototype mouthrinses ACPM and AFPM contained a new sensorial flavor ingredient and were evaluated for comparison against the commerically available reference mouthrinse LCM. The antiplaque effects of the AFPM were not as strong as the alcohol-containing mouthrinses (Table 2) in this study. This result can be attributed to differences in the AFPM formulation, likely affecting EO solubility and the excipient’s ability to impact the EOs’ bactericidal action; however, this effect is not likely due to the absence of alcohol. Moreover, previous laboratory studies demonstrated that much higher concentrations of alcohol are needed to exhibit antimicrobial effects [40]. Despite reduced plaque efficacy, the AFPM showed a strong clinical reduction in gingivitis (Table 2), implicating a mechanism outside of plaque control.

SMS increased the number of bacteria identified at the species level compared to 16S sequencing. This enabled mapping of identified species to their source (oral vs. extraoral) and oral health associations (commensal, gingivitis, malodor, caries) allowing for the exclusive analysis of oral bacteria. Given the presence of environmental, contaminating, and taxonomically undefined species in the metagenomic data [41], bacterial species mapping offered a higher level of specificity during analysis, permitting examination of treatment effects on clinically relevant groups of bacteria (Fig. 4). The absolute abundance measures provided by this technique allowed precise examination of the plaque microbiome composition, foregoing the drawbacks of relative abundance. For example, the total quantity of bacteria in a sample could be reduced, while their relative abundances can remain unchanged, or substantial changes in the relative abundance of one taxon can artificially inflate or deflate the remaining taxa in that sample [13, 42]. The results become even more confounding when individual sample relative abundances are averaged for group comparisons, which has a significant potential for generating misleading information. Absolute bacterial quantification is less prone to misinterpretation, leading to more meaningful comparisons between the clinical sample groups. This is also demonstrated by our data that conform to historically published results, using selective culture-based, colony-counting, and 16S rRNA qPCR methods (Table 4). The quantitative results in this study showed that the microbiota of subjects with moderate gingivitis had greater abundances of commensal and pathogenic species than the healthy cohort (Table 5). In future investigations, in-depth analysis of host inflammatory response may provide a more comprehensive understanding of disease progression.

To minimize inter-subject variability, subjects with gingivitis underwent dental prophylaxis after baseline plaque sampling. Microbiological assessment after prophylaxis and first rinse demonstrated reductions in bacterial load, richness, and diversity with no differences between treatment groups (Fig. 2). This result showed that dental prophylaxis had a substantial initial impact; however, neither of these interventions eradicated all supragingival plaque bacteria. Previously published clinical studies using colony counting further support that EO-containing mouthrinses cannot impact the entirety of the oral microbiome [34–36, 43]. Instead, predominantly early colonizing, health-associated commensals reestablished plaque after repeated use (Fig. 4; Table 5). Dimensional reduction analysis further corroborated that both LCM and ACPM shifted the gingivitis-associated microbiome towards the health-associated microbiome after 4 weeks of use (Fig. 5). In contrast, the microbiome of HA control subjects returned to baseline dysbiosis (Fig. 5). This striking result is the first clear demonstration that LCM, when used twice daily for 4 weeks, can revert a gingivitis-associated microbiome towards a healthier state, suggesting a reset of the plaque microbiome towards an early-colonizing state.

## Conclusions

By utilizing a quantitative method for microbiome analysis, this study provides a level of clarity and sensitivity not achievable by the widely used 16S sequencing approach. The results of this study reinforce the known clinical efficacy of LCM for reducing plaque and gingivitis; additionally, we show that its microbiological action is not due to selective killing of pathogenic bacteria but rather via a reset mechanism, in which the plaque microbiome composition is shifted to a healthier state after repeated use.

### Supplementary Information

Supplementary Material 1.

## Data Availability

Shotgun metagenomic sequence data and sample metadata information are available in the NCBI BioProject database under accession number PRJNA952827. Bacterial classification data are provided in additional file 1.

## Abbreviations

ACPM: Alcohol-containing prototype mouthrinse
AFPM: Alcohol-free prototype mouthrinse
CMUs: Calculated microbial units
EBI: Expanded Gingival Bleeding Index
EO: Essential oil
HA: Hydroalcohol
HOMD: Human Oral Microbiome Database
LCM: LISTERINE® Cool Mint Antiseptic
MGI: The Modified Gingival Index
OST: Oral soft tissue evaluation
PPDs: Periodontal pocket depths
qPCR: Quantitative polymerase chain reaction
SMS: Shotgun metagenomic sequencing
TML: Total microbial load
TPI: Turesky modification of the Quigly-Hein Plaque Index
