# Supplementary material for: Quantitative analysis of the effects of essential oil mouthrinses on clinical plaque microbiome: a parallel-group, randomized trial
Source: BMC Oral Health. 2024 May 18;24:578. doi: 10.1186/s12903-024-04365-9 (PMC11102605; doi:10.1186/s12903-024-04365-9)
Supplement: Supplementary file 1 — Supplementary Material 1. [file 12903_2024_4365_MOESM1_ESM.docx]

**List of bacterial species mapped to clinical relevance**

| Name | TaxID | Genome | Cavities | Gingivitis | Periodontitis | Malodor | Commensal | Pathogenic | Type | Heathy | Unknown | Contaminant | PMID | PMC |
| --- | --- | --- | --- | --- | --- | --- | --- | --- | --- | --- | --- | --- | --- | --- |
| Eubacterium brachy | 35517 | 1.54452 | No | Yes | Yes | No | No | Yes | Opportunistic | No | No | No | 3475328, 3624445 | 269265 |
| Eubacterium infirmum | 56774 | 1.90896 | No | No | No | No | Yes | No |  | No | No | No | 8863423 |  |
| Eubacterium nodatum | 35518 | 1.82956 | No | Yes | Yes | Yes | No | Yes | Opportunistic | No | No | No | 3475328, 3624445, 1299802 | 269265 |
| Eubacterium saphenum | 51123 | 1.95142 | No | Yes | Yes | Yes | No | No |  | No | No | No | 26416306, 30909081, 26511188 | 2863426 |
| Eubacterium sulci | 143393 | 1.73402 |  | Yes | Yes | Yes |  |  |  |  | No | No |  |  |
| Eubacterium yurii | 39498 | 2.53252 |  |  | Yes | Yes |  |  |  |  | No | No |  |  |
| Hallella seregens | 52229 | 3.26675 |  | Yes | Yes |  |  |  |  |  | No | No |  |  |
| Abiotrophia defectiva | 46125 | 2.04344 |  |  |  |  | Yes |  |  |  | No | No |  |  |
| Acidipropionibacterium acidipropionici | 1748 | 3.63868 | Yes |  |  |  |  |  |  |  | No | No |  |  |
| Actinobaculum sp. oral taxon 183 | 712888 | 2.76896 |  |  | Yes |  |  |  |  |  | No | No |  |  |
| Actinomyces cardiffensis | 181487 | 2.22272 |  |  |  |  |  | Yes | Opportunistic |  | No | No |  |  |
| Actinomyces dentalis | 272548 | 3.53139 |  | Yes | Yes |  |  |  |  |  | No | No |  |  |
| Actinomyces georgiae | 52768 | 2.49802 |  |  |  |  | Yes |  |  |  | No | No |  |  |
| Actinomyces gerencseriae | 52769 | 3.42002 |  |  |  |  | Yes |  |  |  | No | No | 9495607 |  |
| Actinomyces graevenitzii | 55565 | 2.10786 |  |  |  |  | Yes |  |  |  | No | No |  |  |
| Actinomyces israelii | 1659 | 3.29939 |  | Yes |  |  |  | Yes | Opportunistic |  | No | No | 9495607 |  |
| Actinomyces johnsonii | 544581 | 3.35268 |  |  |  |  | Yes |  |  |  | No | No |  |  |
| Actinomyces massiliensis | 461393 | 3.39374 |  |  |  |  | Yes |  |  |  | No | No |  |  |
| Actinomyces naeslundii | 1655 | 3.14776 |  |  |  |  | Yes |  |  |  | No | No | 9495607 |  |
| Actinomyces odontolyticus | 1660 | 2.39396 |  |  |  | Yes | Yes |  |  |  | No | No | 9495607, 16091443 |  |
| Actinomyces oris | 544580 | 3.1092 |  |  |  |  | Yes |  |  |  | No | No |  |  |
| Actinomyces slackii | 52774 | 3.1958 |  |  |  |  | Yes |  |  |  | No | No |  |  |
| Actinomyces timonensis | 1288391 | 2.93294 |  |  |  |  | Yes |  |  |  | No | No |  |  |
| Actinomyces viscosus | 1656 | 3.33638 |  |  |  |  | Yes |  |  |  | No | No |  |  |
| Aggregatibacter actinomycetemcomitans | 714 | 2.12758 |  |  | Yes |  |  | Yes | Infectious |  | No | No | 25862077, 25139407 | 4495649, 4213353 |
| Aggregatibacter aphrophilus | 732 | 2.33287 |  |  |  |  | Yes |  |  |  | No | No | 9495607 |  |
| Aggregatibacter segnis | 739 | 1.96566 |  |  |  |  | Yes |  |  |  | No | No |  |  |
| Alloprevotella rava | 671218 | 2.59387 |  |  |  |  | Yes |  |  |  | No | No |  |  |
| Alloprevotella tannerae | 76122 | 2.58572 |  |  |  |  | Yes |  |  |  | No | No |  |  |
| Alloscardovia omnicolens | 419015 | 1.79242 |  |  |  |  | Yes |  |  |  | No | No |  |  |
| Anaeroglobus geminatus | 156456 | 1.79885 |  |  | Yes |  |  |  |  |  | No | No | 22170420 | 3358035 |
| Atopobium parvulum | 1382 | 1.53584 |  |  |  | Yes | Yes |  |  |  | No | No |  |  |
| Atopobium rimae | 1383 | 1.62811 |  |  |  |  | Yes |  |  |  | No | No |  |  |
| Bacteroides thetaiotaomicron | 818 | 6.37285 |  |  |  |  | Yes |  |  |  | No | No |  |  |
| Bacteroides uniformis | 820 | 4.92178 |  |  |  |  | Yes |  |  |  | No | No |  |  |
| Bacteroidetes oral taxon 274 | 652708 | 2.11354 |  |  |  |  | Yes |  |  |  | No | No |  |  |
| Bifidobacterium dentium | 1689 | 2.62554 | Yes |  |  |  |  |  |  |  | No | No |  |  |
| Bulleidia extructa | 118748 | 1.41958 |  |  |  |  |  | Yes | Opportunistic |  | No | No |  |  |
| Campylobacter concisus | 199 | 1.94143 |  |  |  |  | Yes |  |  |  | No | No |  |  |
| Campylobacter curvus | 200 | 1.97126 |  |  |  |  | Yes |  |  |  | No | No |  |  |
| Campylobacter gracilis | 824 | 2.2685 |  |  |  |  | Yes |  |  |  | No | No |  |  |
| Campylobacter rectus | 203 | 2.50223 |  |  | Yes |  |  |  |  |  | No | No |  |  |
| Campylobacter showae | 204 | 2.18735 |  |  | Yes |  |  |  |  |  | No | No |  |  |
| candidate division SR1 bacterium MGEHA | 1293577 | 1.10971 |  |  |  | Yes |  |  |  |  | No | No | 23509275 | 3619370 |
| candidate division TM7 single-cell isolate TM7a | 447454 | 2.86436 |  |  |  |  | Yes | No |  |  | No | No | 30894042 | 6481004 |
| candidate division TM7 single-cell isolate TM7b | 447455 | 0.112753 |  |  |  |  | Yes | No |  |  | No | No | 30894042 | 6481004 |
| candidate division TM7 single-cell isolate TM7c | 447456 | 0.474179 |  |  |  |  | Yes | No |  |  | No | No | 30894042 | 6481004 |
| Candidatus Saccharibacteria oral taxon TM7x | 1476577 | 0.705138 |  |  |  |  | Yes | No |  |  | No | No | 30894042 | 6481004 |
| Capnocytophaga gingivalis | 1017 | 2.75289 |  | Maybe |  |  | Yes | Yes | Opportunistic | No | No | No | 518239 |  |
| Chloroflexi bacterium oral taxon 439 | 712934 | 1.16002 |  |  |  |  | Yes | No |  | No | No | No | 26764907 | 4713201 |
| Corynebacterium durum | 61592 | 3.4016 | No | No | No | No | Yes | No |  | No | No | No | 26811460, 32020052 | 4760785, 7174362 |
| Corynebacterium matruchotii | 43768 | 2.85599 | No | No | No | No | Yes | No |  | No | No | No | 264880 |  |
| Cryptobacterium curtum | 84163 | 1.6178 |  | Yes | Yes | Yes | No | Yes | Infectious | No | No | No | 28979069, 10425779, 17064396 | 5621177, |
| Cutibacterium acnes | 1747 | 2.50237 |  |  |  |  | Yes | Yes | Opportunistic | No | No | No | 26856712 | 5069318 |
| Desulfobulbus sp. oral taxon 041 | 712258 | 2.70424 |  |  |  |  | Yes |  |  |  | No | No | 22170420, 23555659 | 3358035, 3608642 |
| Dialister invisus | 218538 | 1.89596 | Yes | Yes | Yes | Yes | No | Yes | Opportunistic | No | No | No | 14657126, 27219464 | 5122475 |
| Dialister micraerophilus | 309120 | 1.33883 |  |  |  |  | Yes | Yes | Opportunistic |  | No | No | 16280512 |  |
| Eggerthia catenaformis | 31973 | 1.94516 | Yes | Yes | Yes |  | No | Yes | Opportunistic | No | No | No | 26172397, 31002871 |  |
| Eikenella corrodens | 539 | 2.24314 |  | Yes |  |  |  | Yes | Opportunistic |  | No | No | 9495607 |  |
| Filifactor alocis | 143361 | 1.93101 | No | No | Yes | No | No | Yes | Infectious | No | No | No | 22170420, 25841800 | 3358035, 4485945 |
| Fretibacterium fastidiosum | 651822 | 2.72834 |  |  | Yes |  | No |  |  | No | No | No | 22493171, 26936213 | 42924544 |
| Fusobacterium hwasookii | 1583098 | 2.43583 |  |  | Yes |  | No |  |  | No | No | No | 25257648 |  |
| Fusobacterium nucleatum | 851 | 2.39235 |  | Yes | Yes | Yes | No | Yes | Opportunistic | No | No | No |  |  |
| Fusobacterium periodonticum | 860 | 2.54631 |  |  |  | Yes |  |  |  |  | No | No | 22355729 | 3253589 |
| Gemella haemolysans | 1379 | 1.91619 |  | Yes |  |  | No | Yes | Opportunistic | No | No | No | 9574693, 22837552 | 104816, 3420397 |
| Gemella morbillorum | 29391 | 1.75693 |  |  |  |  | Yes | Yes | Opportunistic | No | No | No | 22170420, 9495607 | 3358035 |
| Gemella sanguinis | 84135 | 1.79661 |  |  |  |  | Yes | Yes | Opportunistic | No | No | No | 23520516 | 3592792 |
| Granulicatella adiacens | 46124 | 1.94717 |  |  |  |  | Yes | Yes | Opportunistic |  | No | No | 11154413, 10405400, 29716699, 25139407 | 85281, 5993913, 4213353 |
| Granulicatella elegans | 137732 | 1.74336 |  |  |  |  | Yes | Yes | Opportunistic |  | No | No | 11154413, 10405400 | 85281 |
| Haemophilus haemolyticus | 726 | 1.91514 |  |  |  |  | Yes | No |  | No | No | No | 17687018 | 2045313 |
| Haemophilus influenzae | 727 | 1.8477 |  |  |  |  | Yes | Yes | Opportunistic | No | No | No | 17687018 | 2045313 |
| Haemophilus parahaemolyticus | 735 | 2.09403 |  |  |  |  | Yes | Yes | Opportunistic | No | No | No | 24696434 | 3993099 |
| Haemophilus parainfluenzae | 729 | 2.07915 |  |  |  |  | Yes | Yes | Opportunistic | No | No | No | 6500711 | 261613 |
| Haemophilus paraphrohaemolyticus | 736 | 2.02121 |  |  |  |  | Yes | Yes | Opportunistic | No | No | No | 24696434 | 3993099 |
| Haemophilus pittmaniae | 249188 | 2.18261 |  |  |  |  | Yes | Yes | Opportunistic | No | No | No | 24696434 | 3993099 |
| Haemophilus sputorum | 1078480 | 2.08011 |  |  |  |  | Yes | Yes | Opportunistic | No | No | No | 22336150 |  |
| Johnsonella ignava | 43995 | 2.68708 |  |  |  |  | Yes | Yes | Opportunistic | No | No | No | 22817758, 30642137 | 3507910, 6352272 |
| Kingella denitrificans | 502 | 2.22045 |  |  |  |  | Yes | Yes | Opportunistic |  | No | No | 25821962 | 4378984 |
| Kingella oralis | 505 | 2.40667 |  |  |  |  | Yes | No |  | No | No | No | 9467377 |  |
| Lachnoanaerobaculum saburreum | 467210 | 3.07409 |  |  |  |  | Yes | No |  | No | No | No | 22228654 | 3541795 |
| Lactobacillus casei | 1582 | 2.98937 | Yes |  |  |  | Yes | No |  | Yes | No | No | 25758458 | 4547204 |
| Lactobacillus fermentum | 1613 | 2.01183 | Yes | No | No |  | Yes | Yes | Opportunistic | Yes | No | No | 25758458 | 4547204 |
| Lactobacillus gasseri | 1596 | 1.91731 | Yes | No | No |  | Yes | Yes | Opportunistic | Yes | No | No | 25758458 | 4547204 |
| Lactobacillus oris | 1632 | 2.11599 | No | No | No |  | Yes | No |  | Yes | No | No | 25758458 | 4547204 |
| Lactobacillus paracasei | 1597 | 3.00005 | No | No | No |  | Yes | No |  | Yes | No | No | 20502929 | 3133768 |
| Lactobacillus rhamnosus | 47715 | 2.949 | Yes | No | No |  | Yes | Yes | Opportunistic | Yes | No | No | 25758458 | 4547204 |
| Lactobacillus salivarius | 1624 | 1.98355 | Yes |  |  |  | Yes | No |  | Yes | No | No | 25758458 | 4547204 |
| Lactobacillus vaginalis | 1633 | 1.8786 |  |  |  |  | Yes | No |  |  | No | No | 25758458 | 4547204 |
| Lautropia mirabilis | 47671 | 3.16197 |  |  |  |  | Yes | No |  |  | No | No | 8075812 |  |
| Leptotrichia buccalis | 40542 | 2.46561 |  | Yes |  |  |  | Yes | Opportunistic | No | No | No | 29666288 | 5904416 |
| Leptotrichia goodfellowii | 157692 | 2.28728 |  | Yes |  |  | Yes | Yes | Opportunistic | No | No | No | 29081911 | 5646626 |
| Leptotrichia hofstadii | 157688 | 2.56074 |  |  |  |  | Yes | No |  | No | No | No | 15023979 |  |
| Leptotrichia shahii | 157691 | 2.15289 |  | Yes |  |  |  | No |  |  | No | No | 15023979 |  |
| Leptotrichia trevisanii | 109328 | 2.85336 |  |  |  |  | Yes | Yes | Opportunistic |  | No | No | 30547754 | 6295021 |
| Leptotrichia wadei | 157687 | 2.36941 |  |  |  |  | Yes | Yes | Opportunistic |  | No | No | 15023979 |  |
| Megasphaera micronuciformis | 187326 | 1.76553 |  |  | Yes | Yes |  | No |  |  | No | No | 22355729, 17021095 | 3253589, 1594761 |
| Mitsuokella sp. oral taxon 131 | 1321780 | 2.22845 |  |  | Yes |  |  | Yes | Infectious | No | No | No | 7478766 |  |
| Mogibacterium sp. CM50 | 936375 | 1.88852 |  | Yes | Yes |  |  | Yes | Infectious | No | No | No | 22057871, 29339824 | 3255620, 5932080 |
| Mogibacterium timidum | 35519 | 1.80624 |  | Yes | Yes |  |  | Yes | Infectious | No | No | No | 24031909 | 3768883 |
| Morococcus cerebrosus | 1056807 | 2.4482 |  |  |  |  | Yes | Yes | Opportunistic | No | No | No | 29378989 |  |
| Mycoplasma salivarium | 2124 | 1.22541 |  | Yes |  |  |  | Yes | Opportunistic | No | No | No | 4254176, 3711294 | 268787 |
| Neisseria bacilliformis | 267212 | 2.4 |  |  |  |  | Yes | Yes | Opportunistic | No | No | No | 16455901 | 1392657 |
| Neisseria cinerea | 483 | 1.8745 |  |  |  |  | Yes | Yes | Opportunistic | No | No | No | 6361062 | 270980 |
| Neisseria elongata | 495 | 2.39785 |  |  |  |  | Yes | Yes | Opportunistic | No | No | No | 25814039, 5488467 |  |
| Neisseria gonorrhoeae | 485 | 2.14504 |  |  |  |  | Yes | Yes | Opportunistic | No | No | No | 28369241, 24562188 | 3969750 |
| Neisseria lactamica | 486 | 2.18548 |  |  |  |  | Yes | Yes | Opportunistic | No | No | No | 31959912, 27572971 | 6971049 |
| Neisseria macacae | 496 | 2.74837 |  |  |  |  | Yes | Yes | Opportunistic | No | No | No | 24097834 | 3799226 |
| Neisseria meningitidis | 487 | 2.13097 |  |  |  |  | Yes | Yes | Opportunistic | No | No | No | 19464092 | 2719693 |
| Neisseria mucosa | 488 | 2.51694 |  |  |  |  | Yes | Yes | Opportunistic | No | No | No | 22798652 | 3709538 |
| Neisseria polysaccharea | 489 | 2.09084 |  |  |  |  | Yes | No |  | No | No | No |  |  |
| Neisseria sicca | 490 | 2.52135 |  |  |  |  | Yes | No |  | No | No | No | 27572971, 773308 | 169827 |
| Neisseria subflava | 28449 | 2.26242 |  |  |  |  | Yes | Yes | Opportunistic | No | No | No | 25814039 |  |
| Olsenella profusa | 138595 | 2.72493 | Yes | Yes | Yes |  |  | No |  | No | No | No | 11594611 |  |
| Olsenella uli | 133926 | 2.15324 |  | Yes | Yes |  |  | Yes | Infectious | No | No | No | 11594611 |  |
| Oribacterium asaccharolyticum | 1501332 | 2.52058 |  | Yes |  |  |  | No |  | No | No | No | 24824639, 29988721 | 4129163, 6032013 |
| Oribacterium parvum | 1501329 | 2.47639 |  |  |  |  | Yes | No |  | No | No | No | 24824639 | 4129163 |
| Oribacterium sinus | 237576 | 2.70695 |  |  |  |  | Yes | No |  | No | No | No | 15388717, 17021095 | 1594761 |
| Parascardovia denticolens | 78258 | 1.89145 | Yes |  |  |  |  | No |  | No | No | No | 16707878, 12054242 |  |
| Parvimonas micra | 33033 | 1.6791 |  | Yes | Yes |  |  | Yes | Infectious | No | No | No | 23574465 | 3912758 |
| Peptoanaerobacter stomatis | 796937 | 2.54845 |  |  | Yes |  |  | Yes | Infectious | No | No | No | 31052371, 28438978 | 6630776, 5478963 |
| Peptoniphilus lacrimalis | 33031 | 1.69921 |  |  |  |  | Yes | Yes | Opportunistic | No | No | No | 1390111 |  |
| Peptostreptococcus stomatis | 341694 | 1.98804 |  | Yes | Yes |  |  | Yes | Opportunistic | No | No | No | 16585688, 22413030 | 3295795 |
| Porphyromonas catoniae | 41976 | 2.07083 |  |  |  |  | Yes | No |  | No | No | No | 15583276, 8590687 | 535285 |
| Porphyromonas endodontalis | 28124 | 2.08812 |  |  | Yes | Yes |  | Yes | Infectious | No | No | No | 25139407, 2082242 | 4213353 |
| Porphyromonas gingivalis | 837 | 2.33175 |  |  | Yes | Yes |  | Yes | Infectious | No | No | No | 22170420, 9495607, 15752104, 11083813 | 3358035, 97798 |
| Prevotella aurantiaca | 596085 | 3.00217 |  |  | Yes |  |  | No |  | No | No | No | 19654360 |  |
| Prevotella baroniae | 305719 | 3.11615 |  |  | Yes |  |  | Yes | Opportunistic | No | No | No | 16014480 |  |
| Prevotella bivia | 28125 | 2.49027 |  | Yes | Yes |  |  | Yes | Infectious | No | No | No | 19161595 | 2637877 |
| Prevotella buccae | 28126 | 3.20324 |  |  |  |  | Yes | Yes | Opportunistic | No | No | No | 22684253 |  |
| Prevotella dentalis | 52227 | 3.31834 |  |  |  |  | Yes | Yes | Opportunistic | No | No | No | 28979069 | 5621177 |
| Prevotella denticola | 28129 | 3.05351 |  | Yes | Yes |  | No |  |  | No | No | No | 22170420, 22684253 | 3358035 |
| Prevotella enoeca | 76123 | 2.82273 |  |  |  |  | Yes | No |  | No | No | No | 7981091 |  |
| Prevotella fusca | 589436 | 3.21781 |  |  |  |  | Yes | No |  | No | No | No | 20495041 |  |
| Prevotella histicola | 470565 | 2.99243 | Yes |  |  |  | Yes | No |  | No | No | No |  |  |
| Prevotella intermedia | 28131 | 2.7778 |  | Yes | Yes | Yes |  |  |  | No | No | No | 22170420, 22684253 | 3358035 |
| Prevotella loescheii | 840 | 3.47526 |  | Yes | Yes | Yes |  | Yes | Opportunistic | No | No | No | 1390106, 2082242 |  |
| Prevotella maculosa | 439703 | 3.29642 |  |  |  |  | Yes |  |  | No | No | No | 22684253 |  |
| Prevotella marshii | 189722 | 2.53876 |  |  |  |  | Yes | Yes | Opportunistic | No | No | No | 16014480 |  |
| Prevotella melaninogenica | 28132 | 3.16823 |  |  |  | Yes | Yes |  |  | No | No | No | 22355729, 22684253 | 3253589 |
| Prevotella micans | 189723 | 2.43529 |  |  |  |  | Yes | No |  | No | No | No | 19329604 |  |
| Prevotella multiformis | 282402 | 3.05741 |  |  |  |  | Yes |  |  | No | No | No | 22684253 |  |
| Prevotella multisaccharivorax | 310514 | 3.38864 | Yes |  | Yes |  |  | No |  | No | No | No | 19801230 |  |
| Prevotella nanceiensis | 425941 | 2.64879 |  |  |  |  | Yes | Yes | Opportunistic | No | No | No | 17911286 |  |
| Prevotella nigrescens | 28133 | 2.83797 | Yes |  |  |  | Yes |  |  | No | No | No | 9495607, 22684253 |  |
| Prevotella oralis | 28134 | 2.85737 |  |  |  |  | Yes |  |  | No | No | No | 22684253 |  |
| Prevotella oris | 28135 | 3.16821 |  |  |  |  | Yes |  |  | No | No | No | 22684253 |  |
| Prevotella oulorum | 28136 | 2.82776 |  |  |  |  | Yes |  |  | No | No | No | 22684253 |  |
| Prevotella pallens | 60133 | 3.10871 |  |  |  | Yes |  |  |  | No | No | No | 22355729 | 3253589 |
| Prevotella pleuritidis | 407975 | 2.63504 |  |  |  |  | Yes |  |  | No | No | No | 22684253 |  |
| Prevotella saccharolytica | 633701 | 2.9145 |  |  |  |  | Yes | No |  | No | No | No | 19946051 | 3931283 |
| Prevotella salivae | 228604 | 3.27458 |  |  |  |  | Yes |  |  | No | No | No | 15143039 |  |
| Prevotella scopos | 589437 | 3.20989 |  |  |  |  | Yes | No |  | No | No | No | 20495041 |  |
| Prevotella shahii | 228603 | 3.5007 |  |  |  | Yes | Yes | No |  | No | No | No | 22355729 | 3253589 |
| Prevotella veroralis | 28137 | 2.84575 |  |  |  | Yes | Yes | No |  | No | No | No | 1390106 |  |
| Propionibacterium acidifaciens | 556499 | 3.0439 | Yes |  |  |  |  |  |  | No | No | No | 31254840 |  |
| Pseudopropionibacterium propionicum | 1750 | 3.40512 |  |  | Yes |  |  | Yes | Opportunistic | No | No | No | 31052361 | 6630690 |
| Pseudoramibacter alactolyticus | 113287 | 2.36288 |  |  | Yes |  |  | Yes | Opportunistic | No | No | No | 14651280 |  |
| Pyramidobacter piscolens | 638849 | 2.93121 | Yes |  | Yes |  |  | Yes | Opportunistic | No | No | No | 19406777 | 2868594 |
| Rothia aeria | 172042 | 2.58868 |  |  |  |  | Yes | Yes | Opportunistic | No | No | No | 28082174, 28082174 |  |
| Rothia dentocariosa | 2047 | 2.49282 | Yes |  |  |  | Yes | Yes | Opportunistic | No | No | No | 9495607, 3475328 |  |
| Rothia mucilaginosa | 43675 | 2.28519 | Yes |  |  |  | Yes | Yes | Opportunistic | No | No | No |  |  |
| Scardovia inopinata | 78259 | 1.80093 | Yes |  |  |  |  | Yes | Opportunistic | No | No | No | 12054242 |  |
| Scardovia wiggsiae | 230143 | 1.55316 | Yes |  |  |  |  | No |  | No | No | No | 29104444 | 5665406 |
| Selenomonas artemidis | 671224 | 2.28045 |  |  |  | Yes | Yes | Yes | Opportunistic | No | No | No | 2405009 | 269556 |
| Selenomonas flueggei | 135080 | 2.16606 |  |  |  |  | Yes | No |  | No | No | No | 20831580 |  |
| Selenomonas infelix | 135082 | 2.41363 |  |  |  |  | Yes | Yes | Opportunistic | No | No | No | 2405009 | 269556 |
| Selenomonas noxia | 135083 | 2.0842 |  |  | Yes |  |  | Yes | Infectious | No | No | No | 9495607 |  |
| Selenomonas sputigena | 69823 | 2.56391 |  |  | Yes |  |  | Yes | Infectious | No | No | No | 129552, 27563202 | 4976549 |
| Shuttleworthia satelles | 177972 | 2.16948 |  | Yes | Yes |  |  | Yes | Opportunistic | No | No | No | 12361248 |  |
| Slackia exigua | 84109 | 2.09952 |  | Yes | Yes | Yes |  | Yes | Opportunistic | No | No | No | 28979069 | 5621177 |
| Solobacterium moorei | 102148 | 1.998998 |  |  |  | Yes | Yes | Yes | Opportunistic | No | No | No | 22355729, 21525228 | 3253589, 3147872 |
| Stomatobaculum longum | 796942 | 2.31363 |  |  |  |  | Yes | Yes | Opportunistic | No | No | No | 22843721, 31874981 | 3709536, 6930300 |
| Streptococcus agalactiae | 1311 | 2.08293 |  |  |  |  | Yes | Yes | Opportunistic | No | No | No | 19721085 | 2738137 |
| Streptococcus anginosus | 1328 | 1.95819 |  |  |  |  | Yes | Yes | Opportunistic | No | No | No | 10843047 |  |
| Streptococcus australis | 113107 | 1.96241 |  |  |  |  | Yes | Yes | Opportunistic | No | No | No | 11491323 |  |
| Streptococcus constellatus | 76860 | 1.9139 |  | Yes | Yes |  | Yes | Yes | Opportunistic | No | No | No | 19829816 | 2740169 |
| Streptococcus cristatus | 45634 | 2.07309 |  |  |  |  | Yes | No |  | Yes | No | No | 20826648 | 3020839 |
| Streptococcus gordonii | 1302 | 2.19256 |  |  |  |  | Yes | Yes | Opportunistic | Yes | No | No | 30338752, 26875613 | 6287261 |
| Streptococcus infantis | 68892 | 1.8695 |  |  |  |  | Yes | No |  | No | No | No | 9734047 |  |
| Streptococcus intermedius | 1338 | 1.94241 |  |  |  |  | Yes | Yes | Opportunistic | No | No | No | 9495607, 21738290 | 3124902 |
| Streptococcus massiliensis | 313439 | 1.86415 |  |  |  |  | Yes | No |  | No | No | No | 26782571 |  |
| Streptococcus mitis | 28037 | 2.00271 |  |  |  |  | Yes | Yes | Opportunistic | Yes | No | No | 3475328, 30338752, 26875613 | 6287261 |
| Streptococcus mutans | 1309 | 1.98387 | Yes |  |  |  | Yes | No |  | No | No | No | 9495607, 30338752 | 6287261 |
| Streptococcus oralis | 1303 | 1.96978 |  |  |  |  | Yes | Yes | Opportunistic | Yes | No | No | 9495607, 30338752, 26875613 | 6287261 |
| Streptococcus parasanguinis | 1318 | 2.13514 |  |  |  |  | Yes | Yes | Opportunistic | Yes | No | No | 30338752, 26875613 | 6287261 |
| Streptococcus peroris | 68891 | 1.63992 |  |  |  |  | Yes | No |  | No | No | No | 9734047 |  |
| Streptococcus pneumoniae | 1313 | 2.08598 |  |  |  |  | Yes | Yes | Opportunistic | No | No | No | 10678950 | 97291 |
| Streptococcus pseudopneumoniae | 257758 | 2.17262 |  |  |  |  | Yes | Yes | Opportunistic | No | No | No |  |  |
| Streptococcus pyogenes | 1314 | 1.79141 |  |  |  |  |  | Yes | Infectious | No | No | No | 19721085 | 2738137 |
| Streptococcus salivarius | 1304 | 2.18552 |  |  |  |  | Yes | Yes | Opportunistic | Yes | No | No | 24271166 | 3911234 |
| Streptococcus sanguinis | 1305 | 2.36222 |  |  |  |  | Yes | Yes | Opportunistic | No | No | No | 22170420, 30338752, 26875613 | 3358035, 6287261 |
| Streptococcus sinensis | 176090 | 2.06184 |  |  |  |  | Yes | Yes | Opportunistic | No | No | No | 11880397, 19330895 | 120286 |
| Streptococcus sobrinus | 1310 | 2.11248 | Yes |  |  |  | Yes | No |  | No | No | No | 30338752 | 6287261 |
| Streptococcus vestibularis | 1343 | 1.89801 |  |  |  | Yes | Yes | Yes | Opportunistic | No | No | No | 25983909, 16091443 | 4421209 |
| Tannerella forsythia | 28112 | 3.30018 |  |  | Yes | Yes |  | Yes | Infectious | No | No | No | 9495607, 25139407, 15752104 | 4213353 |
| Treponema denticola | 158 | 2.82537 |  |  | Yes | Yes |  | Yes | Infectious | No | No | No | 22170420, 15752104 | 3358035 |
| Treponema lecithinolyticum | 53418 | 2.34089 |  |  | Yes |  |  | Yes | Infectious | No | No | No | 10555310, 15845368 |  |
| Treponema maltophilum | 51160 | 2.53039 |  |  | Yes |  |  | Yes | Infectious | No | No | No | 8782684 |  |
| Treponema medium | 58231 | 2.72751 |  |  | Yes |  |  | Yes | Infectious | No | No | No | 22170420, 8995804 | 3358035 |
| Treponema putidum | 221027 | 2.78645 |  |  | Yes |  |  | Yes | Infectious | No | No | No | 16238598 |  |
| Treponema socranskii | 53419 | 2.80463 |  |  | Yes |  |  | Yes | Infectious | No | No | No | 3475328 |  |
| Treponema vincentii | 69710 | 2.60404 |  |  | Yes |  |  | Yes | Infectious | No | No | No | 22170420 | 3358035 |
| Veillonella atypica | 39777 | 2.07195 |  |  |  |  | Yes | No |  | No | No | No | 28473967 | 5397411 |
| Veillonella dispar | 39778 | 2.08434 |  |  |  | Yes | Yes | No |  | No | No | No | 28473967, 16091443 | 5397411 |
| Veillonella parvula | 29466 | 2.14419 |  |  |  |  | Yes | Yes | Opportunistic | No | No | No | 9495607, 28473967 | 5397411 |
| Capnocytophaga sp. CM59 | 936370 | 2.66929 |  |  |  |  |  |  |  |  | Yes | No | 22057871 | 3255620 |
| Capnocytophaga sp. oral taxon 324 | 712211 | 2.66929 |  |  |  |  |  |  |  |  | Yes | No |  |  |
| Capnocytophaga sp. oral taxon 326 | 712212 | 2.66929 |  |  |  |  |  |  |  |  | Yes | No |  |  |
| Capnocytophaga sp. oral taxon 329 | 706435 | 2.66929 |  |  |  |  |  |  |  |  | Yes | No |  |  |
| Capnocytophaga sp. oral taxon 332 | 712213 | 2.66929 |  |  |  |  |  |  |  |  | Yes | No |  |  |
| Capnocytophaga sp. oral taxon 335 | 712215 | 2.66929 |  |  |  |  |  |  |  |  | Yes | No |  |  |
| Capnocytophaga sp. oral taxon 336 | 712216 | 2.66929 |  |  |  |  |  |  |  |  | Yes | No |  |  |
| Capnocytophaga sp. oral taxon 338 | 710239 | 2.66929 |  |  |  |  |  |  |  |  | Yes | No |  |  |
| Capnocytophaga sp. oral taxon 380 | 712217 | 2.66929 |  |  |  |  |  |  |  |  | Yes | No |  |  |
| Corynebacterium imitans | 156978 | 2.56546 |  |  |  |  |  | Yes | Infectious | No | No | Yes | 9230366 | 229887 |
| Eubacterium rectale | 39491 | 3.23449 |  |  |  |  |  |  |  |  |  | Yes | 26619944 |  |
| Propionibacterium humerusii | 1050843 | 2.62329 |  |  |  |  |  |  |  |  |  | Yes |  |  |
| Propionibacterium namnetense | 1574624 | 2.38655 |  |  |  |  |  |  |  |  |  | Yes |  |  |
| Pseudomonas geniculata | 86188 | 4.71396 |  |  |  |  |  |  |  |  |  | Yes |  |  |
| Scytonema hofmanni UTEX B 1581 | 379535 | 8.13344 |  |  |  |  |  |  |  |  |  | Yes |  |  |
| Achromobacter insuavis | 1287735 | 6.87612 |  |  |  |  |  |  |  |  |  | Yes |  |  |
| Acinetobacter baumannii | 470 | 3.97473 |  |  |  |  |  |  |  |  |  | Yes |  |  |
| Acinetobacter calcoaceticus/baumannii complex | 909768 | 3.67016 |  |  |  |  |  |  |  |  |  | Yes |  |  |
| Acinetobacter johnsonii | 40214 | 3.56222 |  |  |  |  |  |  |  |  |  | Yes |  |  |
| Acinetobacter junii | 40215 | 3.37031 |  |  |  |  |  |  |  |  |  | Yes |  |  |
| Acinetobacter lwoffii | 28090 | 3.35313 |  |  |  |  |  |  |  |  |  | Yes |  |  |
| Acinetobacter pittii | 48296 | 3.99363 |  |  |  |  |  |  |  |  |  | Yes |  |  |
| Acinetobacter soli | 487316 | 3.47098 |  |  |  |  |  |  |  |  |  | Yes |  |  |
| Acinetobacter ursingii | 108980 | 3.48116 |  |  |  |  |  |  |  |  |  | Yes |  |  |
| Actinobacillus minor | 51047 | 2.27621 |  |  |  |  |  |  |  |  |  | Yes |  |  |
| Actinobacillus pleuropneumoniae | 715 | 2.27448 |  |  |  |  |  |  |  |  |  | Yes |  |  |
| Actinobacillus ureae | 723 | 2.47481 |  |  |  |  |  |  |  |  |  | Yes |  |  |
| Actinomyces neuii | 33007 | 2.3076 |  |  |  |  |  | Yes |  |  |  | Yes |  |  |
| Actinomyces polynesiensis | 1325934 | 2.87845 |  |  |  |  |  |  |  |  |  | Yes |  |  |
| Actinomyces turicensis | 131111 | 1.98876 |  |  |  |  |  |  |  |  |  | Yes |  |  |
| Actinomyces urogenitalis | 103621 | 2.60442 |  |  |  |  |  |  |  |  |  | Yes |  |  |
| Aerococcus viridans | 1377 | 2.00481 |  |  |  |  |  |  |  |  |  | Yes |  |  |
| Aeromonas hydrophila | 644 | 4.93286 |  |  |  |  |  |  |  |  |  | Yes |  |  |
| Akkermansia muciniphila | 239935 | 2.76245 |  |  |  |  |  |  |  |  |  | Yes |  |  |
| Alistipes finegoldii | 214856 | 3.50237 |  |  |  |  |  |  |  |  |  | Yes |  |  |
| Alistipes ihumii | 1470347 | 2.77282 |  |  |  |  |  |  |  |  |  | Yes |  |  |
| Alistipes inops | 1501391 | 2.30482 |  |  |  |  |  |  |  |  |  | Yes |  |  |
| Alistipes obesi | 1118061 | 3.16368 |  |  |  |  |  |  |  |  |  | Yes |  |  |
| Alistipes putredinis | 28117 | 2.37226 |  |  |  |  |  |  |  |  |  | Yes |  |  |
| Anoxybacillus flavithermus | 33934 | 2.77262 |  |  |  |  |  |  |  |  |  | Yes |  |  |
| Atopobium vaginae | 82135 | 1.45072 |  |  |  |  |  |  |  |  |  | Yes |  |  |
| Azospirillum lipoferum | 193 | 7.77069 |  |  |  |  |  |  |  |  |  | Yes |  |  |
| Bacillus cereus | 1396 | 5.75713 |  |  |  |  |  |  |  |  |  | Yes |  |  |
| Bacillus coagulans | 1398 | 3.40527 |  |  |  |  |  |  |  |  |  | Yes |  |  |
| Bacillus subtilis | 1423 | 4.13481 |  |  |  |  |  |  |  |  |  | Yes |  |  |
| Bacillus thermoamylovorans | 35841 | 3.82405 |  |  |  |  |  |  |  |  |  | Yes |  |  |
| Bacteroides caccae | 47678 | 5.38919 |  |  |  |  |  |  |  |  |  | Yes |  |  |
| Bacteroides coprocola | 310298 | 4.13856 |  |  |  |  |  |  |  |  |  | Yes |  |  |
| Bacteroides coprophilus | 387090 | 3.85266 |  |  |  |  |  |  |  |  |  | Yes |  |  |
| Bacteroides dorei | 357276 | 5.44491 |  |  |  |  |  |  |  |  |  | Yes |  |  |
| Bacteroides eggerthii | 28111 | 4.30351 |  |  |  |  |  |  |  |  |  | Yes |  |  |
| Bacteroides finegoldii | 338188 | 5.00461 |  |  |  |  |  |  |  |  |  | Yes |  |  |
| Bacteroides fragilis | 817 | 5.27083 |  |  |  |  |  |  |  |  |  | Yes |  |  |
| Bacteroides massiliensis | 204516 | 4.443 |  |  |  |  |  |  |  |  |  | Yes |  |  |
| Bacteroides ovatus | 28116 | 6.71678 |  |  |  |  |  |  |  |  |  | Yes |  |  |
| Bacteroides stercoris | 46506 | 4.08011 |  |  |  |  |  |  |  |  |  | Yes |  |  |
| Bacteroides vulgatus | 821 | 5.05011 |  |  |  |  |  |  |  |  |  | Yes |  |  |
| Bacteroides xylanisolvens | 371601 | 6.35844 |  |  |  |  |  |  |  |  |  | Yes |  |  |
| Barnesiella intestinihominis | 487174 | 3.1501 |  |  |  |  |  |  |  |  |  | Yes |  |  |
| Bifidobacterium adolescentis | 1680 | 2.20301 |  |  |  |  |  |  |  |  |  | Yes |  |  |
| Bifidobacterium animalis | 28025 | 1.93269 |  |  |  |  |  |  |  |  |  | Yes |  |  |
| Bifidobacterium bifidum | 1681 | 2.20341 |  |  |  |  |  |  |  |  |  | Yes |  |  |
| Bifidobacterium breve | 1685 | 2.33155 |  |  |  |  |  |  |  |  |  | Yes |  |  |
| Bifidobacterium catenulatum | 1686 | 2.08276 |  |  |  |  |  |  |  |  |  | Yes |  |  |
| Bifidobacterium longum | 216816 | 2.39546 |  |  |  |  |  |  |  |  |  | Yes |  |  |
| Bifidobacterium moukalabense | 1333651 | 2.52266 |  |  |  |  |  |  |  |  |  | Yes |  |  |
| Bifidobacterium pseudocatenulatum | 28026 | 2.52266 |  |  |  |  |  |  |  |  |  | Yes |  |  |
| Bifidobacterium pseudolongum | 1694 | 2.01678 |  |  |  |  |  |  |  |  |  | Yes |  |  |
| Bilophila sp. 4_1_30 | 693988 | 4.01374 |  |  |  |  |  |  |  |  |  | Yes |  |  |
| Bilophila wadsworthia | 35833 | 4.39119 |  |  |  |  |  |  |  |  |  | Yes |  |  |
| Bordetella bronchiseptica | 518 | 5.19988 |  |  |  |  |  |  |  |  |  | Yes |  |  |
| Brachybacterium muris | 219301 | 3.25747 |  |  |  |  |  |  |  |  |  | Yes |  |  |
| Brachybacterium paraconglomeratum | 173362 | 3.78416 |  |  |  |  |  |  |  |  |  | Yes |  |  |
| Bradyrhizobium japonicum | 375 | 9.46079 |  |  |  |  |  |  |  |  |  | Yes |  |  |
| Brevibacterium linens | 1703 | 3.92017 |  |  |  |  |  |  |  |  |  | Yes |  |  |
| Brevundimonas naejangsanensis | 588932 | 3.00004 |  |  |  |  |  |  |  |  |  | Yes |  |  |
| Brevundimonas nasdae | 172043 | 3.40783 |  |  |  |  |  |  |  |  |  | Yes |  |  |
| Campylobacter jejuni | 197 | 1.68112 |  |  |  |  |  |  |  |  |  | Yes |  |  |
| Candidatus Endolissoclinum faulkneri | 1263979 | 1.49992 |  |  |  |  |  |  |  |  |  | Yes |  |  |
| Candidatus Fervidibacteria_u_s | 1383058 | 1.05546 |  |  |  |  |  |  |  |  |  | Yes |  |  |
| Candidatus Riesia_u_s | 401618 | 0.534351 |  |  |  |  |  |  |  |  |  | Yes |  |  |
| Candidatus Sulcia muelleri | 336810 | 0.270029 |  |  |  |  |  |  |  |  |  | Yes |  |  |
| Cellulosimicrobium cellulans | 1710 | 4.27511 |  |  |  |  |  |  |  |  |  | Yes |  |  |
| Cellulosimicrobium sp. MM | 1523621 | 4.32197 |  |  |  |  |  |  |  |  |  | Yes |  |  |
| Christensenella minuta | 626937 | 2.94023 |  |  |  |  |  |  |  |  |  | Yes |  |  |
| Citricoccus sp. CH26A | 1045009 | 3.56134 |  |  |  |  |  |  |  |  |  | Yes |  |  |
| Clostridioides difficile | 1496 | 4.15343 |  |  |  |  |  |  |  |  |  | Yes |  |  |
| Comamonas testosteroni | 285 | 5.64935 |  |  |  |  |  |  |  |  |  | Yes |  |  |
| Coprococcus comes | 410072 | 3.3395 |  |  |  |  |  |  |  |  |  | Yes |  |  |
| Coprococcus sp. ART55/1 | 751585 | 2.91054 |  |  |  |  |  |  |  |  |  | Yes |  |  |
| Coriobacteriaceae bacterium BV3Ac1 | 1111135 | 1.80138 |  |  |  |  |  |  |  |  |  | Yes |  |  |
| Corynebacterium accolens | 38284 | 2.46564 |  |  |  |  |  |  |  |  |  | Yes | 26733066 | 4725001 |
| Corynebacterium argentoratense | 42817 | 2.02091 |  |  |  |  |  | Yes | Opportunistic |  |  | Yes | 26933505 | 4765771 |
| Corynebacterium crenatum | 168810 | 3.30773 |  |  |  |  |  |  |  |  |  | Yes |  |  |
| Corynebacterium falsenii | 108486 | 2.38178 |  |  |  |  |  |  |  |  |  | Yes |  |  |
| Corynebacterium genitalium | 38288 | 2.33364 |  |  |  |  |  |  |  |  |  | Yes |  |  |
| Corynebacterium ihumii | 1232427 | 2.25128 |  |  |  |  |  |  |  |  |  | Yes | 25197488 | 4149009 |
| Corynebacterium jeddahense | 1414719 | 2.47213 |  |  |  |  |  |  |  |  |  | Yes | 25197478 | 4149016 |
| Corynebacterium jeikeium | 38289 | 2.41465 |  |  |  |  |  | Yes | Opportunistic |  |  | Yes |  |  |
| Corynebacterium kroppenstedtii | 161879 | 2.51532 |  |  |  |  |  |  |  |  |  | Yes |  |  |
| Corynebacterium lipophiloflavum | 161889 | 2.38578 |  |  |  |  |  |  |  |  |  | Yes |  |  |
| Corynebacterium minutissimum | 38301 | 2.69597 |  |  |  |  |  | Yes | Infectious |  |  | Yes |  |  |
| Corynebacterium propinquum | 43769 | 2.55312 |  |  |  |  |  |  |  |  |  | Yes |  |  |
| Corynebacterium pseudogenitalium | 38303 | 2.60143 |  |  |  |  |  |  |  |  |  | Yes |  |  |
| Corynebacterium tuberculostearicum | 38304 | 2.37226 |  |  |  |  |  |  |  |  |  | Yes |  |  |
| Corynebacterium urealyticum | 43771 | 2.36922 |  |  |  |  |  |  |  |  |  | Yes |  |  |
| Corynebacterium variabile | 1727 | 3.18913 |  |  |  |  |  |  |  |  |  | Yes |  |  |
| Cutibacterium avidum | 33010 | 2.54063 |  |  |  |  |  |  |  |  |  | Yes |  |  |
| Cutibacterium granulosum | 33011 | 2.13429 |  |  |  |  |  |  |  |  |  | Yes |  |  |
| Dehalococcoides mccartyi | 61435 | 1.38891 |  |  |  |  |  |  |  |  |  | Yes |  |  |
| Delftia acidovorans | 80866 | 6.54935 |  |  |  |  |  |  |  |  |  | Yes |  |  |
| Dermacoccus nishinomiyaensis | 1274 | 3.09805 |  |  |  |  |  |  |  |  |  | Yes |  |  |
| Desulfovibrio desulfuricans | 876 | 3.25144 |  |  |  |  |  |  |  |  |  | Yes |  |  |
| Dorea longicatena | 88431 | 3.12661 |  |  |  |  |  |  |  |  |  | Yes |  |  |
| Enhydrobacter aerosaccus | 225324 | 6.76976 |  |  |  |  |  |  |  |  |  | Yes |  |  |
| Enterobacter cloacae | 550 | 4.95909 |  |  |  |  |  |  |  |  |  | Yes |  |  |
| Enterobacter cloacae complex | 354276 | 4.95909 |  |  |  |  |  |  |  |  |  | Yes |  |  |
| Enterococcus faecalis | 1351 | 2.96863 |  |  |  |  |  |  |  |  |  | Yes |  |  |
| Enterococcus faecium | 1352 | 2.9207 |  |  |  |  |  | Yes | Infectious |  |  | Yes |  |  |
| Enterorhabdus caecimuris | 671266 | 2.88737 |  |  |  |  |  |  |  |  |  | Yes |  |  |
| Enterorhabdus mucosicola | 580026 | 2.94802 |  |  |  |  |  |  |  |  |  | Yes |  |  |
| Escherichia coli | 562 | 5.14165 |  |  |  |  | Yes | Yes | Opportunistic | No |  | Yes |  |  |
| Ewingella americana | 41202 | 4.94177 |  |  |  |  |  | Yes | Infectious |  |  | Yes |  |  |
| Faecalibacterium prausnitzii | 853 | 2.9698 |  |  |  |  |  |  |  |  |  | Yes |  |  |
| Finegoldia magna | 1260 | 1.8999 |  |  |  |  |  |  |  |  |  | Yes |  |  |
| Glaesserella parasuis | 738 | 2.22147 |  |  |  |  |  |  |  |  |  | Yes |  |  |
| Glycomyces tenuis | 58116 | 5.88399 |  |  |  |  |  |  |  |  |  | Yes |  |  |
| Gordonia bronchialis | 2054 | 5.29896 |  |  |  |  |  |  |  |  |  | Yes |  |  |
| Gordonia terrae | 2055 | 5.6733 |  |  |  |  |  |  |  |  |  | Yes |  |  |
| Gracilibacteria bacterium JGI 0000069-P22 | 1130343 | 0.341011 |  |  |  |  |  |  |  |  |  | Yes |  |  |
| Haemophilus aegyptius | 197575 | 1.92618 |  |  |  |  |  | Yes | Opportunistic |  |  | Yes |  |  |
| Halorubrum distributum | 29283 | 3.30613 |  |  |  |  |  |  |  |  |  | Yes |  |  |
| Hassallia byssoidea | 482630 | 7.50152 |  |  |  |  |  |  |  |  |  | Yes |  |  |
| Inquilinus limosus | 171674 | 7.41371 |  |  |  |  |  |  |  |  |  | Yes |  |  |
| Janibacter hoylei | 364298 | 3.12251 |  |  |  |  |  |  |  |  |  | Yes |  |  |
| Janthinobacterium lividum | 29581 | 6.37032 |  |  |  |  |  |  |  |  |  | Yes |  |  |
| Kingella kingae | 504 | 1.99286 |  |  |  |  |  |  |  |  |  | Yes |  |  |
| Klebsiella aerogenes | 548 | 5.26068 |  |  |  |  |  |  |  |  |  | Yes |  |  |
| Klebsiella michiganensis | 1134687 | 6.1921 |  |  |  |  |  |  |  |  |  | Yes |  |  |
| Klebsiella oxytoca | 571 | 6.04904 |  |  |  |  |  |  |  |  |  | Yes |  |  |
| Klebsiella pneumoniae | 573 | 5.58728 |  |  |  |  |  |  |  |  |  | Yes |  |  |
| Kocuria palustris | 71999 | 2.84394 |  |  |  |  |  |  |  |  |  | Yes |  |  |
| Kocuria polaris | 136273 | 3.80676 |  |  |  |  |  |  |  |  |  | Yes |  |  |
| Kocuria rhizophila | 72000 | 2.70162 |  |  |  |  |  |  |  |  |  | Yes |  |  |
| Kytococcus sedentarius | 1276 | 2.61891 |  |  |  |  |  |  |  |  |  | Yes |  |  |
| Lachnospira eligens | 39485 | 3.03579 |  |  |  |  |  |  |  |  |  | Yes |  |  |
| Lactobacillus antri | 227943 | 2.24184 |  |  |  |  |  |  |  |  |  | Yes |  |  |
| Lactobacillus crispatus | 47770 | 2.16396 |  |  |  |  |  |  |  |  |  | Yes |  |  |
| Lactobacillus curvatus | 28038 | 1.93262 |  |  |  |  |  |  |  |  |  | Yes |  |  |
| Lactobacillus delbrueckii | 1584 | 1.87507 |  |  |  |  |  |  |  |  |  | Yes |  |  |
| Lactobacillus gastricus | 227942 | 1.90487 |  |  |  |  |  |  |  |  |  | Yes |  |  |
| Lactobacillus helveticus | 1587 | 2.05871 |  |  |  |  |  |  |  |  |  | Yes |  |  |
| Lactobacillus johnsonii | 33959 | 1.91814 |  |  |  |  |  |  |  |  |  | Yes |  |  |
| Lactobacillus mucosae | 97478 | 2.0791 |  |  |  |  |  |  |  |  |  | Yes |  |  |
| Lactobacillus murinus | 1622 | 2.39145 |  |  |  |  |  |  |  |  |  | Yes |  |  |
| Lactobacillus plantarum | 1590 | 3.24481 |  |  |  |  |  |  |  |  |  | Yes |  |  |
| Lactobacillus reuteri | 1598 | 2.11531 |  |  |  |  |  |  |  |  |  | Yes |  |  |
| Lactobacillus sakei | 1599 | 1.99257 |  |  |  |  |  |  |  |  |  | Yes |  |  |
| Lactobacillus ultunensis | 227945 | 2.24841 |  |  |  |  |  |  |  |  |  | Yes |  |  |
| Lactococcus garvieae | 1363 | 2.05489 |  |  |  |  |  |  |  |  |  | Yes |  |  |
| Lactococcus lactis | 1358 | 2.50288 |  |  |  |  |  |  |  |  |  | Yes |  |  |
| Leclercia adecarboxylata | 83655 | 4.6076 |  |  |  |  |  |  |  |  |  | Yes |  |  |
| Leptolyngbya boryana | 1184 | 7.01857 |  |  |  |  |  |  |  |  |  | Yes |  |  |
| Leucobacter chironomi | 491918 | 2.96471 |  |  |  |  |  |  |  |  |  | Yes |  |  |
| Leuconostoc lactis | 1246 | 1.72068 |  |  |  |  |  |  |  |  |  | Yes |  |  |
| Leuconostoc mesenteroides | 1245 | 1.90636 |  |  |  |  |  |  |  |  |  | Yes |  |  |
| Massilia timonae | 47229 | 5.30535 |  |  |  |  |  |  |  |  |  | Yes |  |  |
| Meiothermus ruber | 277 | 3.02929 |  |  |  |  |  |  |  |  |  | Yes |  |  |
| Methylobacterium populi | 223967 | 5.77461 |  |  |  |  |  |  |  |  |  | Yes |  |  |
| Methylobacterium sp. EUR3 AL-11 | 1305730 | 5.50554 |  |  |  |  |  |  |  |  |  | Yes |  |  |
| Methylobacterium sp. UNCCL110 | 1449057 | 5.50554 |  |  |  |  |  |  |  |  |  | Yes |  |  |
| Microbacterium laevaniformans | 36807 | 3.11559 |  |  |  |  |  |  |  |  |  | Yes |  |  |
| Microbacterium sp. KROCY2 | 1305732 | 3.52618 |  |  |  |  |  |  |  |  |  | Yes |  |  |
| Micrococcus luteus | 1270 | 2.5013 |  |  |  |  |  |  |  |  |  | Yes |  |  |
| Mobiluncus curtisii | 2051 | 2.15297 |  |  |  |  |  |  |  |  |  | Yes |  |  |
| Mycobacterium iranicum | 912594 | 6.33565 |  |  |  |  |  |  |  |  |  | Yes |  |  |
| Mycobacterium tuberculosis | 1773 | 4.38305 |  |  |  |  |  |  |  |  |  | Yes |  |  |
| Mycoplasma hominis | 2098 | 0.679518 |  |  |  |  |  |  |  |  |  | Yes |  |  |
| Negativicoccus succinicivorans | 620903 | 1.45809 |  |  |  |  |  |  |  |  |  | Yes |  |  |
| Neisseria shayeganii | 607712 | 2.35455 |  |  |  |  |  |  |  |  |  | Yes |  |  |
| Ochrobactrum anthropi | 529 | 4.90444 |  |  |  |  |  |  |  |  |  | Yes |  |  |
| Oxalobacter formigenes | 847 | 2.46777 |  |  |  |  |  |  |  |  |  | Yes |  |  |
| Paenibacillus sophorae | 1333845 | 5.80703 |  |  |  |  |  |  |  |  |  | Yes |  |  |
| Parabacteroides distasonis | 823 | 5.1561 |  |  |  |  |  |  |  |  |  | Yes |  |  |
| Paracoccus sanguinis | 1545044 | 3.49404 |  |  |  |  |  |  |  |  |  | Yes |  |  |
| Paraprevotella clara | 454154 | 4.2105 |  |  |  |  |  |  |  |  |  | Yes |  |  |
| Pasteurella bettyae | 752 | 2.29808 |  |  |  |  |  |  |  |  |  | Yes |  |  |
| Pectobacterium carotovorum | 554 | 4.82476 |  |  |  |  |  |  |  |  |  | Yes |  |  |
| Peptoniphilus rhinitidis | 1175452 | 1.88526 |  |  |  |  |  |  |  |  |  | Yes |  |  |
| Peptostreptococcus anaerobius | 1261 | 2.09502 |  |  |  |  |  |  |  |  |  | Yes |  |  |
| Phocaeicola abscessus | 555313 | 2.53688 |  |  |  |  |  |  |  |  |  | Yes |  |  |
| Pilimelia anulata | 53371 | 6.98001 |  |  |  |  |  |  |  |  |  | Yes |  |  |
| Porphyromonas asaccharolytica | 28123 | 2.19275 |  |  |  |  |  |  |  |  |  | Yes |  |  |
| Porphyromonas gulae | 111105 | 2.35611 |  |  |  |  |  |  |  |  |  | Yes |  |  |
| Porphyromonas macacae | 28115 | 2.32457 |  |  |  |  |  |  |  |  |  | Yes |  |  |
| Porphyromonas uenonis | 281920 | 2.25441 |  |  |  |  |  |  |  |  |  | Yes |  |  |
| Prevotella amnii | 419005 | 2.39194 |  |  |  |  |  |  |  |  |  | Yes |  |  |
| Prevotella bergensis | 242750 | 3.27367 |  |  |  |  |  |  |  |  |  | Yes |  |  |
| Prevotella copri | 165179 | 3.67025 |  |  |  |  |  |  |  |  |  | Yes |  |  |
| Prevotella disiens | 28130 | 2.80264 |  |  |  |  |  |  |  |  |  | Yes |  |  |
| Prevotella falsenii | 515414 | 2.80024 |  |  |  |  |  |  |  |  |  | Yes |  |  |
| Prevotella timonensis | 386414 | 3.1164 |  |  |  |  |  |  |  |  |  | Yes |  |  |
| Propionibacterium freudenreichii | 1744 | 2.63087 |  |  |  |  |  |  |  |  |  | Yes |  |  |
| Pseudomonas aeruginosa | 287 | 6.60808 |  |  |  |  |  |  |  |  |  | Yes |  |  |
| Pseudomonas alcaligenes | 43263 | 4.6293 |  |  |  |  |  |  |  |  |  | Yes |  |  |
| Pseudomonas fluorescens | 294 | 6.39182 |  |  |  |  |  |  |  |  |  | Yes |  |  |
| Pseudomonas fragi | 296 | 5.0723 |  |  |  |  |  |  |  |  |  | Yes |  |  |
| Pseudomonas monteilii | 76759 | 5.78828 |  |  |  |  |  |  |  |  |  | Yes |  |  |
| Pseudomonas simiae | 321846 | 6.20446 |  |  |  |  |  |  |  |  |  | Yes |  |  |
| Pseudomonas stutzeri group | 136846 | 4.34292 |  |  |  |  |  |  |  |  |  | Yes |  |  |
| Ralstonia pickettii | 329 | 5.25477 |  |  |  |  |  |  |  |  |  | Yes |  |  |
| Rhodococcus qingshengii | 334542 | 6.77944 |  |  |  |  |  |  |  |  |  | Yes |  |  |
| Rhodopseudomonas palustris | 1076 | 5.39916 |  |  |  |  |  |  |  |  |  | Yes |  |  |
| Roseburia intestinalis | 166486 | 4.29467 |  |  |  |  |  |  |  |  |  | Yes |  |  |
| Roseomonas aerilata | 452982 | 6.42847 |  |  |  |  |  |  |  |  |  | Yes |  |  |
| Roseomonas cervicalis | 204525 | 5.10098 |  |  |  |  |  |  |  |  |  | Yes |  |  |
| Rubellimicrobium mesophilum | 1123067 | 4.92184 |  |  |  |  |  |  |  |  |  | Yes |  |  |
| Rubellimicrobium thermophilum | 295419 | 3.16314 |  |  |  |  |  |  |  |  |  | Yes |  |  |
| Rubritepida flocculans | 182403 | 3.83412 |  |  |  |  |  |  |  |  |  | Yes |  |  |
| Rudanella lutea | 451374 | 6.54604 |  |  |  |  |  |  |  |  |  | Yes |  |  |
| Ruminococcus bicirculans | 1160721 | 2.49455 |  |  |  |  |  |  |  |  |  | Yes |  |  |
| Ruminococcus bromii | 40518 | 2.17731 |  |  |  |  |  |  |  |  |  | Yes |  |  |
| Ruminococcus callidus | 40519 | 3.02711 |  |  |  |  |  |  |  |  |  | Yes |  |  |
| Ruminococcus lactaris | 46228 | 2.83359 |  |  |  |  |  |  |  |  |  | Yes |  |  |
| Salmonella enterica | 28901 | 4.79856 |  |  |  |  |  |  |  |  |  | Yes |  |  |
| Selenomonas bovis | 416586 | 2.1601 |  |  |  |  |  |  |  |  |  | Yes |  |  |
| Senegalimassilia anaerobia | 1473216 | 2.38105 |  |  |  |  |  |  |  |  |  | Yes |  |  |
| Serinicoccus profundi | 1078471 | 3.4548 |  |  |  |  |  |  |  |  |  | Yes |  |  |
| Serratia marcescens | 615 | 5.21014 |  |  |  |  |  |  |  |  |  | Yes |  |  |
| Serratia symbiotica | 138074 | 2.43681 |  |  |  |  |  |  |  |  |  | Yes |  |  |
| Simonsiella muelleri | 72 | 2.42124 |  |  |  |  |  |  |  |  |  | Yes |  |  |
| Solimonas variicoloris | 254408 | 4.11945 |  |  |  |  |  |  |  |  |  | Yes |  |  |
| Sphingobium yanoikuyae | 13690 | 5.45963 |  |  |  |  |  |  |  |  |  | Yes |  |  |
| Sphingomonas astaxanthinifaciens | 407019 | 2.53303 |  |  |  |  |  |  |  |  |  | Yes |  |  |
| Sphingomonas melonis | 152682 | 3.99628 |  |  |  |  |  |  |  |  |  | Yes |  |  |
| Staphylococcus aureus | 1280 | 2.83686 |  |  |  |  |  |  |  |  |  | Yes |  |  |
| Staphylococcus capitis | 29388 | 2.48502 |  |  |  |  |  |  |  |  |  | Yes |  |  |
| Staphylococcus epidermidis | 1282 | 2.5166 |  |  |  |  |  |  |  |  |  | Yes |  |  |
| Staphylococcus haemolyticus | 1283 | 2.5086 |  |  |  |  |  |  |  |  |  | Yes |  |  |
| Staphylococcus hominis | 1290 | 2.25 |  |  |  |  |  |  |  |  |  | Yes |  |  |
| Staphylococcus pettenkoferi | 170573 | 2.47232 |  |  |  |  |  |  |  |  |  | Yes |  |  |
| Staphylococcus pseudintermedius | 283734 | 2.65741 |  |  |  |  |  |  |  |  |  | Yes |  |  |
| Staphylococcus xylosus | 1288 | 2.83592 |  |  |  |  |  |  |  |  |  | Yes |  |  |
| Stenotrophomonas maltophilia | 40324 | 4.63645 |  |  |  |  |  |  |  |  |  | Yes |  |  |
| Streptococcus caballi | 439220 | 2.12204 |  |  |  |  |  |  |  |  |  | Yes |  |  |
| Streptococcus castoreus | 254786 | 1.88354 |  |  |  |  |  |  |  |  |  | Yes |  |  |
| Streptococcus downei | 1317 | 2.23576 |  |  |  |  |  |  |  |  |  | Yes |  |  |
| Streptococcus dysgalactiae | 1334 | 2.16398 |  |  |  |  |  |  |  |  |  | Yes |  |  |
| Streptococcus equi | 1336 | 2.12988 |  |  |  |  |  |  |  |  |  | Yes |  |  |
| Streptococcus suis | 1307 | 2.0972 |  |  |  |  |  |  |  |  |  | Yes |  |  |
| Streptococcus thermophilus | 1308 | 1.83195 |  |  |  |  |  |  |  |  |  | Yes |  |  |
| Sutterella wadsworthensis | 40545 | 2.87582 |  |  |  |  |  |  |  |  |  | Yes |  |  |
| Tessaracoccus massiliensis | 1522311 | 3.21171 |  |  |  |  |  |  |  |  |  | Yes |  |  |
| Thermus scotoductus | 37636 | 2.31096 |  |  |  |  |  |  |  |  |  | Yes |  |  |
| Thiobacillus denitrificans | 36861 | 3.18934 |  |  |  |  |  |  |  |  |  | Yes |  |  |
| Varibaculum cambriense | 184870 | 2.09699 |  |  |  |  |  |  |  |  |  | Yes |  |  |
| Variovorax paradoxus | 34073 | 7.01173 |  |  |  |  |  |  |  |  |  | Yes |  |  |
| Vibrio cholerae | 666 | 4.02008 |  |  |  |  |  |  |  |  |  | Yes |  |  |
| Weissella cibaria | 137591 | 2.45564 |  |  |  |  |  |  |  |  |  | Yes |  |  |
| Weissella confusa | 1583 | 2.25848 |  |  |  |  |  |  |  |  |  | Yes |  |  |
| Yaniella halotolerans | 225453 | 2.77546 |  |  |  |  |  |  |  |  |  | Yes |  |  |
| Zymomonas mobilis | 542 | 2.06692 |  |  |  |  |  |  |  |  |  | Yes |  |  |
| Acetomicrobium_u_s | 49894 | 2.02102 |  |  |  |  |  |  |  |  | Yes |  |  |  |
| Acidaminococcus_u_s | 904 | 2.33707 |  |  |  |  |  |  |  |  | Yes |  |  |  |
| Acidimicrobiaceae_u_s | 84994 | 2.08252 |  |  |  |  |  |  |  |  | Yes |  |  |  |
| Acidipropionibacterium_u_s | 1912215 | 3.17172 |  |  |  |  |  |  |  |  | Yes |  |  |  |
| Acidithiobacillales_u_s | 225057 | 3.01542 |  |  |  |  |  |  |  |  | Yes |  |  |  |
| Acidovorax_u_s | 12916 | 5.0149 |  |  |  |  |  |  |  |  | Yes |  |  |  |
| Acinetobacter sp. 479375 | 1310601 | 3.67016 |  |  |  |  |  |  |  |  | Yes |  |  |  |
| Acinetobacter sp. NIPH 284 | 1217704 | 3.67016 |  |  |  |  |  |  |  |  | Yes |  |  |  |
| Acinetobacter_u_s | 469 | 3.67016 |  |  |  |  |  |  |  |  | Yes |  |  |  |
| Actibacterium_u_s | 1433986 | 2.70858 |  |  |  |  |  |  |  |  | Yes |  |  |  |
| Actinobacillus_u_s | 713 | 2.32916 |  |  |  |  |  |  |  |  | Yes |  |  |  |
| actinobacterium SCGC AAA023-D18 | 932037 | 0.753259 |  |  |  |  |  |  |  |  | Yes |  |  |  |
| actinobacterium SCGC AAA027-J17 | 932040 | 0.966562 |  |  |  |  |  |  |  |  | Yes |  |  |  |
| Actinokineospora_u_s | 39845 | 6.43951 |  |  |  |  |  |  |  |  | Yes |  |  |  |
| Actinomadura_u_s | 1988 | 9.06353 |  |  |  |  |  |  |  |  | Yes |  |  |  |
| Actinomyces sp. oral taxon 170 | 712117 | 2.40388 |  |  |  |  |  |  |  |  | Yes |  |  |  |
| Actinomyces sp. oral taxon 171 | 706438 | 2.40388 |  |  |  |  |  |  |  |  | Yes |  |  |  |
| Actinomyces sp. oral taxon 175 | 712119 | 2.40388 |  |  |  |  |  |  |  |  | Yes |  |  |  |
| Actinomyces sp. oral taxon 178 | 710238 | 2.40388 |  |  |  |  |  |  |  |  | Yes |  |  |  |
| Actinomyces sp. oral taxon 180 | 651609 | 2.40388 |  |  |  |  |  |  |  |  | Yes |  |  |  |
| Actinomyces sp. oral taxon 448 | 712124 | 2.40388 |  |  |  |  |  |  |  |  | Yes |  |  |  |
| Actinomyces sp. oral taxon 848 | 649739 | 2.40388 |  |  |  |  |  |  |  |  | Yes |  |  |  |
| Actinomyces sp. oral taxon 849 | 653385 | 2.40388 |  |  |  |  |  |  |  |  | Yes |  |  |  |
| Actinomyces sp. oral taxon 877 | 1227263 | 2.40388 |  |  |  |  |  |  |  |  | Yes |  |  |  |
| Actinoplanes_u_s | 1865 | 9.24001 |  |  |  |  |  |  |  |  | Yes |  |  |  |
| Actinotalea_u_s | 458839 | 4.02736 |  |  |  |  |  |  |  |  | Yes |  |  |  |
| Actinotignum_u_s | 1653174 | 2.074915 |  |  |  |  |  |  |  |  | Yes |  |  |  |
| Aerococcus_u_s | 1375 | 2.10637 |  |  |  |  |  |  |  |  | Yes |  |  |  |
| Aeromonas_u_s | 642 | 4.6156 |  |  |  |  |  |  |  |  | Yes |  |  |  |
| Aggregatibacter sp. oral taxon 458 | 712148 | 2.01662 |  |  |  |  |  |  |  |  | Yes |  |  |  |
| Aggregatibacter_u_s | 416916 | 2.01662 |  |  |  |  |  |  |  |  | Yes |  |  |  |
| Alcaligenes_u_s | 507 | 4.27369 |  |  |  |  |  |  |  |  | Yes |  |  |  |
| Alistipes sp. HGB5 | 908612 | 2.50162 |  |  |  |  |  |  |  |  | Yes |  |  |  |
| Alistipes_u_s | 239759 | 2.50162 |  |  |  |  |  |  |  |  | Yes |  |  |  |
| Alloscardovia_u_s | 419014 | 1.96101 |  |  |  |  |  |  |  |  | Yes |  |  |  |
| alpha proteobacterium LLX12A | 1229484 | 5.9611 |  |  |  |  |  |  |  |  | Yes |  |  |  |
| alpha proteobacterium SCGC AAA023-L09 | 938615 | 0.774923 |  |  |  |  |  |  |  |  | Yes |  |  |  |
| alpha proteobacterium SCGC AAA028-D10 | 938641 | 0.925141 |  |  |  |  |  |  |  |  | Yes |  |  |  |
| Aminobacterium_u_s | 81466 | 1.66661 |  |  |  |  |  |  |  |  | Yes |  |  |  |
| Amycolatopsis_u_s | 1813 | 9.33291 |  |  |  |  |  |  |  |  | Yes |  |  |  |
| Anaerobutyricum hallii | 39488 | 3.42094 |  |  |  |  |  |  |  |  | Yes |  |  |  |
| Anaerococcus prevotii | 33034 | 2.00181 |  |  |  |  |  |  |  |  | Yes |  |  |  |
| Anaeromyxobacter_u_s | 161492 | 5.16981 |  |  |  |  |  |  |  |  | Yes |  |  |  |
| Anoxybacillus_u_s | 150247 | 3.54109 |  |  |  |  |  |  |  |  | Yes |  |  |  |
| Aquabacterium sp. NJ1 | 1538295 | 4.20412 |  |  |  |  |  |  |  |  | Yes |  |  |  |
| Aquificaceae_u_s | 64898 | 1.60136 |  |  |  |  |  |  |  |  | Yes |  |  |  |
| Arcanobacterium_u_s | 28263 | 1.94117 |  |  |  |  |  |  |  |  | Yes |  |  |  |
| Arenimonas_u_s | 490567 | 2.68096 |  |  |  |  |  |  |  |  | Yes |  |  |  |
| Arthrobacter_u_s | 1663 | 4.39917 |  |  |  |  |  |  |  |  | Yes |  |  |  |
| Asaia_u_s | 91914 | 3.74303 |  |  |  |  |  |  |  |  | Yes |  |  |  |
| Atopobium sp. BS2 | 936550 | 1.97242 |  |  |  |  |  |  |  |  | Yes |  |  |  |
| Atopobium sp. ICM42b | 1190620 | 1.97242 |  |  |  |  |  |  |  |  | Yes |  |  |  |
| Atopobium sp. oral taxon 199 | 712156 | 1.97242 |  |  |  |  |  |  |  |  | Yes |  |  |  |
| Atopobium sp. oral taxon 810 | 712158 | 1.97242 |  |  |  |  |  |  |  |  | Yes |  |  |  |
| Aureimonas_u_s | 414371 | 4.84506 |  |  |  |  |  |  |  |  | Yes |  |  |  |
| Azoarcus_u_s | 12960 | 4.82525 |  |  |  |  |  |  |  |  | Yes |  |  |  |
| Azonexaceae_u_s | 2008795 | 3.623615 |  |  |  |  |  |  |  |  | Yes |  |  |  |
| Azospirillum_u_s | 191 | 6.98287 |  |  |  |  |  |  |  |  | Yes |  |  |  |
| Bacillus sp. EGD-AK10 | 1386080 | 4.94364 |  |  |  |  |  |  |  |  | Yes |  |  |  |
| Bacillus_u_s | 1386 | 4.94364 |  |  |  |  |  |  |  |  | Yes |  |  |  |
| Bacteroides sp. 3_1_40A | 469593 | 4.56992 |  |  |  |  |  |  |  |  | Yes |  |  |  |
| Bacteroides sp. 4_1_36 | 457393 | 4.56992 |  |  |  |  |  |  |  |  | Yes |  |  |  |
| Bacteroides sp. 4_3_47FAA | 457394 | 4.56992 |  |  |  |  |  |  |  |  | Yes |  |  |  |
| Bacteroides sp. 9_1_42FAA | 457395 | 4.56992 |  |  |  |  |  |  |  |  | Yes |  |  |  |
| Bacteroides_u_s | 816 | 4.56992 |  |  |  |  |  |  |  |  | Yes |  |  |  |
| Bacteroidetes bacterium oral taxon 272 | 651591 | 2.50274 |  |  |  |  |  |  |  |  | Yes |  |  |  |
| Belnapia sp. F-4-1 | 1545443 | 5.96228 |  |  |  |  |  |  |  |  | Yes |  |  |  |
| Bifidobacterium sp. MSTE12 | 1161409 | 1.96646 |  |  |  |  |  |  |  |  | Yes |  |  |  |
| Blastocatellia_u_s | 1562566 | 4.396284 |  |  |  |  |  |  |  |  | Yes |  |  |  |
| Blastococcus_u_s | 38501 | 4.33786 |  |  |  |  |  |  |  |  | Yes |  |  |  |
| Blastomonas_u_s | 150203 | 4.10593 |  |  |  |  |  |  |  |  | Yes |  |  |  |
| Bordetella_u_s | 517 | 4.53739 |  |  |  |  |  |  |  |  | Yes |  |  |  |
| Brachyspira_u_s | 29521 | 2.4584 |  |  |  |  |  |  |  |  | Yes |  |  |  |
| Bradyrhizobium sp. DFCI-1 | 1230476 | 8.04555 |  |  |  |  |  |  |  |  | Yes |  |  |  |
| Bradyrhizobium_u_s | 374 | 8.04555 |  |  |  |  |  |  |  |  | Yes |  |  |  |
| Brevundimonas_u_s | 41275 | 3.09879 |  |  |  |  |  |  |  |  | Yes |  |  |  |
| Brochothrix_u_s | 2755 | 2.459205 |  |  |  |  |  |  |  |  | Yes |  |  |  |
| Burkholderia_u_s | 32008 | 7.56994 |  |  |  |  |  |  |  |  | Yes |  |  |  |
| Calothrix sp. PCC 7103 | 32057 | 7.5123 |  |  |  |  |  |  |  |  | Yes |  |  |  |
| Campylobacter sp. 10_1_50 | 665939 | 1.67047 |  |  |  |  |  |  |  |  | Yes |  |  |  |
| Campylobacter sp. FOBRC14 | 936554 | 1.67047 |  |  |  |  |  |  |  |  | Yes |  |  |  |
| Campylobacter_u_s | 194 | 1.67047 |  |  |  |  |  |  |  |  | Yes |  |  |  |
| Capnocytophaga_u_s | 1016 | 2.66929 |  |  |  |  |  |  |  |  | Yes |  |  |  |
| Cardiobacterium_u_s | 2717 | 3.86333 |  |  |  |  |  |  |  |  | Yes |  |  |  |
| Carnobacterium_u_s | 2747 | 2.04768 |  |  |  |  |  |  |  |  | Yes |  |  |  |
| Caulobacter_u_s | 75 | 4.88028 |  |  |  |  |  |  |  |  | Yes |  |  |  |
| Cellulosimicrobium_u_s | 157920 | 4.32197 |  |  |  |  |  |  |  |  | Yes |  |  |  |
| Christensenella_u_s | 990721 | 2.72439 |  |  |  |  |  |  |  |  | Yes |  |  |  |
| Chromobacterium_u_s | 535 | 4.80715 |  |  |  |  |  |  |  |  | Yes |  |  |  |
| Chrysiogenaceae_u_s | 189770 | 2.87677 |  |  |  |  |  |  |  |  | Yes |  |  |  |
| Citrobacter_u_s | 544 | 5.04594 |  |  |  |  |  |  |  |  | Yes |  |  |  |
| Clavibacter_u_s | 1573 | 3.11185 |  |  |  |  |  |  |  |  | Yes |  |  |  |
| Cohnella_u_s | 329857 | 6.42625 |  |  |  |  |  |  |  |  | Yes |  |  |  |
| Conchiformibius_u_s | 334107 | 2.188915 |  |  |  |  |  |  |  |  | Yes |  |  |  |
| Coprothermobacter_u_s | 68335 | 1.60805 |  |  |  |  |  |  |  |  | Yes |  |  |  |
| Corynebacterium sp. KPL1824 | 1203561 | 2.4825 |  |  |  |  |  |  |  |  | Yes |  |  |  |
| Cupriavidus_u_s | 106589 | 6.76618 |  |  |  |  |  |  |  |  | Yes |  |  |  |
| Delftia_u_s | 80865 | 6.47613 |  |  |  |  |  |  |  |  | Yes |  |  |  |
| Dermacoccus sp. Ellin185 | 188626 | 3.07499 |  |  |  |  |  |  |  |  | Yes |  |  |  |
| Dermacoccus_u_s | 57495 | 3.07499 |  |  |  |  |  |  |  |  | Yes |  |  |  |
| Desulfobulbus sp. Dsb5 | 1292025 | 2.70424 |  |  |  |  |  |  |  |  | Yes |  |  |  |
| Desulfobulbus_u_s | 893 | 2.70424 |  |  |  |  |  |  |  |  | Yes |  |  |  |
| Desulfomicrobium_u_s | 898 | 3.31012 |  |  |  |  |  |  |  |  | Yes |  |  |  |
| Desulfovibrio sp. Dsv1 | 1292024 | 3.06467 |  |  |  |  |  |  |  |  | Yes |  | 22170420, 23555659 | 3358035, 3608642 |
| Desulfovibrio_u_s | 872 | 3.06467 |  |  |  |  |  |  |  |  | Yes |  |  |  |
| Dialister_u_s | 39948 | 1.91727 |  |  |  |  |  |  |  |  | Yes |  |  |  |
| Dictyoglomus_u_s | 13 | 1.86743 |  |  |  |  |  |  |  |  | Yes |  |  |  |
| Dietzia_u_s | 37914 | 3.72031 |  |  |  |  |  |  |  |  | Yes |  |  |  |
| Dorea_u_s | 189330 | 2.9208 |  |  |  |  |  |  |  |  | Yes |  |  |  |
| Elizabethkingia_u_s | 308865 | 4.04858 |  |  |  |  |  |  |  |  | Yes |  |  |  |
| Enterobacter_u_s | 547 | 4.90297 |  |  |  |  |  |  |  |  | Yes |  |  |  |
| Enterococcus_u_s | 1350 | 2.9768 |  |  |  |  |  |  |  |  | Yes |  |  |  |
| Enterorhabdus_u_s | 580024 | 2.79295 |  |  |  |  |  |  |  |  | Yes |  |  |  |
| Escherichia_u_s | 561 | 4.83642 |  |  |  |  |  |  |  |  | Yes |  |  |  |
| Exiguobacterium_u_s | 33986 | 2.9343 |  |  |  |  |  |  |  |  | Yes |  |  |  |
| Facklamia_u_s | 66831 | 2.07512 |  |  |  |  |  |  |  |  | Yes |  |  |  |
| Flammeovirga_u_s | 59739 | 7.72505 |  |  |  |  |  |  |  |  | Yes |  |  |  |
| Flavihumibacter_u_s | 1004301 | 4.43832 |  |  |  |  |  |  |  |  | Yes |  |  |  |
| Flavobacterium_u_s | 237 | 3.918 |  |  |  |  |  |  |  |  | Yes |  |  |  |
| Fusobacterium sp. CM1 | 936561 | 2.12718 |  |  |  |  |  |  |  |  | Yes |  |  |  |
| Fusobacterium sp. CM21 | 936562 | 2.12718 |  |  |  |  |  |  |  |  | Yes |  |  |  |
| Fusobacterium sp. CM22 | 936563 | 2.12718 |  |  |  |  |  |  |  |  | Yes |  |  |  |
| Fusobacterium sp. OBRC1 | 1032505 | 2.12718 |  |  |  |  |  |  |  |  | Yes |  |  |  |
| Fusobacterium sp. oral taxon 370 | 712288 | 2.12718 |  |  |  |  |  |  |  |  | Yes |  |  |  |
| Fusobacterium_u_s | 848 | 2.12718 |  |  |  |  |  |  |  |  | Yes |  |  |  |
| Gallibacterium_u_s | 155493 | 2.453674 |  |  |  |  |  |  |  |  | Yes |  |  |  |
| Gemella_u_s | 1378 | 1.76968 |  |  |  |  |  |  |  |  | Yes |  |  |  |
| Gemmatimonas_u_s | 173479 | 3.85651 |  |  |  |  |  |  |  |  | Yes |  |  |  |
| Geobacter_u_s | 28231 | 3.55123 |  |  |  |  |  |  |  |  | Yes |  |  |  |
| Gloeobacter_u_s | 33071 | 4.53346 |  |  |  |  |  |  |  |  | Yes |  |  |  |
| Glycomyces_u_s | 58113 | 5.11627 |  |  |  |  |  |  |  |  | Yes |  |  |  |
| Gramella_u_s | 292691 | 3.48162 |  |  |  |  |  |  |  |  | Yes |  |  |  |
| Granulicatella_u_s | 117563 | 1.92747 |  |  |  |  |  |  |  |  | Yes |  |  |  |
| Haemophilus sp. oral taxon 851 | 762964 | 1.97402 |  |  |  |  |  |  |  |  | Yes |  |  |  |
| Haemophilus_u_s | 724 | 1.97402 |  |  |  |  |  |  |  |  | Yes |  |  |  |
| Halanaerobium_u_s | 2330 | 2.51233 |  |  |  |  |  |  |  |  | Yes |  |  |  |
| Halococcus_u_s | 2249 | 3.87336 |  |  |  |  |  |  |  |  | Yes |  |  |  |
| Haloferax sp. ATCC BAA-644 | 1227462 | 3.83076 |  |  |  |  |  |  |  |  | Yes |  |  |  |
| Haloferax sp. ATCC BAA-645 | 1227463 | 3.83076 |  |  |  |  |  |  |  |  | Yes |  |  |  |
| Halomonas_u_s | 2745 | 3.83061 |  |  |  |  |  |  |  |  | Yes |  |  |  |
| Helicobacter_u_s | 209 | 1.87917 |  |  |  |  |  |  |  |  | Yes |  |  |  |
| Herbaspirillum_u_s | 963 | 5.55286 |  |  |  |  |  |  |  |  | Yes |  |  |  |
| Holospora_u_s | 44747 | 1.43033 |  |  |  |  |  |  |  |  | Yes |  |  |  |
| Hylemonella gracilis | 80880 | 3.82161 |  |  |  |  |  |  |  |  | Yes |  |  |  |
| Hymenobacter aerophilus | 119644 | 4.25304 |  |  |  |  |  |  |  |  | Yes |  |  |  |
| Hymenobacter sp. APR13 | 1356852 | 5.02391 |  |  |  |  |  |  |  |  | Yes |  |  |  |
| Idiomarina_u_s | 135575 | 2.33587 |  |  |  |  |  |  |  |  | Yes |  |  |  |
| Ilumatobacter_u_s | 682522 | 2.44059 |  |  |  |  |  |  |  |  | Yes |  |  |  |
| Janibacter_u_s | 53457 | 3.63784 |  |  |  |  |  |  |  |  | Yes |  |  |  |
| Janthinobacterium sp. RA13 | 1502762 | 6.31217 |  |  |  |  |  |  |  |  | Yes |  |  |  |
| Janthinobacterium_u_s | 29580 | 6.31217 |  |  |  |  |  |  |  |  | Yes |  |  |  |
| Jonesia_u_s | 43673 | 2.89389 |  |  |  |  |  |  |  |  | Yes |  |  |  |
| Kibdelosporangium_u_s | 2029 | 11.7523 |  |  |  |  |  |  |  |  | Yes |  |  |  |
| Kingella_u_s | 32257 | 1.70204 |  |  |  |  |  |  |  |  | Yes |  |  |  |
| Klebsiella_u_s | 570 | 5.46791 |  |  |  |  |  |  |  |  | Yes |  |  |  |
| Kocuria sp. UCD-OTCP | 1292021 | 2.84283 |  |  |  |  |  |  |  |  | Yes |  |  |  |
| Kocuria_u_s | 57493 | 2.84283 |  |  |  |  |  |  |  |  | Yes |  |  |  |
| Kribbella_u_s | 182639 | 8.57728 |  |  |  |  |  |  |  |  | Yes |  |  |  |
| Ktedonobacteria_u_s | 388447 | 3.30132 |  |  |  |  |  |  |  |  | Yes |  |  |  |
| Kurthia_u_s | 1649 | 3.10372 |  |  |  |  |  |  |  |  | Yes |  |  |  |
| Kutzneria_u_s | 43356 | 11.6465 |  |  |  |  |  |  |  |  | Yes |  |  |  |
| Lachnoanaerobaculum sp. ICM7 | 936594 | 2.77996 |  |  |  |  |  |  |  |  | Yes |  |  |  |
| Lachnoanaerobaculum sp. MSX33 | 936596 | 2.87105 |  |  |  |  |  |  |  |  | Yes |  |  |  |
| Lachnoanaerobaculum sp. OBRC5-5 | 936595 | 2.2809 |  |  |  |  |  |  |  |  | Yes |  |  |  |
| Lachnoanaerobaculum_u_s | 1164882 | 2.2809 |  |  |  |  |  |  |  |  | Yes |  |  |  |
| Lachnospiraceae bacterium AC2012 | 1392494 | 2.55544 |  |  |  |  |  |  |  |  | Yes |  |  |  |
| Lachnospiraceae bacterium oral taxon 082 | 712976 | 2.94584 |  |  |  |  |  |  |  |  | Yes |  |  |  |
| Lachnospiraceae bacterium oral taxon 500 | 712991 | 3.24474 |  |  |  |  |  |  |  |  | Yes |  |  |  |
| Lactobacillus_u_s | 1578 | 2.34877 |  |  |  |  |  |  |  |  | Yes |  |  |  |
| Lactococcus_u_s | 1357 | 2.29745 |  |  |  |  |  |  |  |  | Yes |  |  |  |
| Leptospira_u_s | 171 | 4.00239 |  |  |  |  |  |  |  |  | Yes |  |  |  |
| Leptotrichia sp. oral taxon 215 | 712359 | 2.35428 |  |  |  |  |  |  |  |  | Yes |  |  |  |
| Leptotrichia sp. oral taxon 225 | 671213 | 2.35428 |  |  |  |  |  |  |  |  | Yes |  |  |  |
| Leptotrichia sp. oral taxon 879 | 1227267 | 2.35428 |  |  |  |  |  |  |  |  | Yes |  |  |  |
| Leptotrichia_u_s | 32067 | 2.35428 |  |  |  |  |  |  |  |  | Yes |  |  |  |
| Leuconostoc_u_s | 1243 | 1.63335 |  |  |  |  |  |  |  |  | Yes |  |  |  |
| Limnohabitans_u_s | 665874 | 3.38359 |  |  |  |  |  |  |  |  | Yes |  |  |  |
| Luteimonas_u_s | 83614 | 3.40161 |  |  |  |  |  |  |  |  | Yes |  |  |  |
| Lysobacter_u_s | 68 | 4.01898 |  |  |  |  |  |  |  |  | Yes |  |  |  |
| Marinobacterium_u_s | 48075 | 4.0308 |  |  |  |  |  |  |  |  | Yes |  |  |  |
| Marmoricola_u_s | 86795 | 4.10826 |  |  |  |  |  |  |  |  | Yes |  |  |  |
| Massilia_u_s | 149698 | 6.02061 |  |  |  |  |  |  |  |  | Yes |  |  |  |
| Megamonas_u_s | 158846 | 2.29196 |  |  |  |  |  |  |  |  | Yes |  |  |  |
| Megasphaera sp. BV3C16-1 | 1111454 | 2.17601 |  |  |  |  |  |  |  |  | Yes |  |  |  |
| Megasphaera_u_s | 906 | 2.17601 |  |  |  |  |  |  |  |  | Yes |  |  |  |
| Mesorhizobium_u_s | 68287 | 6.63908 |  |  |  |  |  |  |  |  | Yes |  |  |  |
| Methylibium_u_s | 316612 | 4.61633 |  |  |  |  |  |  |  |  | Yes |  |  |  |
| Methylobacterium_u_s | 407 | 5.50554 |  |  |  |  |  |  |  |  | Yes |  |  |  |
| Methylophilus_u_s | 16 | 2.94034 |  |  |  |  |  |  |  |  | Yes |  |  |  |
| Methylopila_u_s | 61653 | 4.40213 |  |  |  |  |  |  |  |  | Yes |  |  |  |
| Methylotenera_u_s | 359407 | 1.81 |  |  |  |  |  |  |  |  | Yes |  |  |  |
| Methyloversatilis_u_s | 378210 | 2.88326 |  |  |  |  |  |  |  |  | Yes |  |  |  |
| Microbacterium_u_s | 33882 | 3.52618 |  |  |  |  |  |  |  |  | Yes |  |  |  |
| Microvirga_u_s | 186650 | 4.7281 |  |  |  |  |  |  |  |  | Yes |  |  |  |
| Mitsuokella_u_s | 52225 | 2.22845 |  |  |  |  |  |  |  |  | Yes |  |  |  |
| Mobilicoccus_u_s | 984996 | 3.44978 |  |  |  |  |  |  |  |  | Yes |  |  |  |
| Mobiluncus_u_s | 2050 | 2.291816 |  |  |  |  |  |  |  |  | Yes |  |  |  |
| Modestobacter_u_s | 88138 | 4.84339 |  |  |  |  |  |  |  |  | Yes |  |  |  |
| Mogibacterium_u_s | 86331 | 1.88852 |  |  |  |  |  |  |  |  | Yes |  |  |  |
| Moraxella_u_s | 475 | 2.11178 |  |  |  |  |  |  |  |  | Yes |  |  |  |
| Moritella_u_s | 58050 | 4.83642 |  |  |  |  |  |  |  |  | Yes |  |  |  |
| Mycobacterium_u_s | 1763 | 5.61673 |  |  |  |  |  |  |  |  | Yes |  |  |  |
| Nakamurella_u_s | 53460 | 4.71495 |  |  |  |  |  |  |  |  | Yes |  |  |  |
| Neisseria sp. GT4A_CT1 | 665946 | 2.31438 |  |  |  |  |  |  |  |  | Yes |  |  |  |
| Neisseria sp. oral taxon 014 | 641148 | 2.31438 |  |  |  |  |  |  |  |  | Yes |  |  |  |
| Neisseria sp. oral taxon 020 | 712401 | 2.31438 |  |  |  |  |  |  |  |  | Yes |  |  |  |
| Neisseria_u_s | 482 | 2.31438 |  |  |  |  |  |  |  |  | Yes |  |  |  |
| Neorickettsia_u_s | 33993 | 0.859205 |  |  |  |  |  |  |  |  | Yes |  |  |  |
| Nesterenkonia_u_s | 57494 | 2.92613 |  |  |  |  |  |  |  |  | Yes |  |  |  |
| Nitrobacter_u_s | 911 | 3.79403 |  |  |  |  |  |  |  |  | Yes |  |  |  |
| Nitrosococcus_u_s | 1227 | 3.76583 |  |  |  |  |  |  |  |  | Yes |  |  |  |
| Nocardioides_u_s | 1839 | 4.37803 |  |  |  |  |  |  |  |  | Yes |  |  |  |
| Nocardiopsis_u_s | 2013 | 6.20939 |  |  |  |  |  |  |  |  | Yes |  |  |  |
| Novosphingobium_u_s | 165696 | 4.62698 |  |  |  |  |  |  |  |  | Yes |  |  |  |
| Ochrobactrum_u_s | 528 | 4.83434 |  |  |  |  |  |  |  |  | Yes |  |  |  |
| Olsenella sp. oral taxon 809 | 661086 | 2.14077 |  |  |  |  |  |  |  |  | Yes |  |  |  |
| Olsenella_u_s | 133925 | 2.14077 |  |  |  |  |  |  |  |  | Yes |  |  |  |
| Oribacterium sp. oral taxon 078 | 652706 | 2.24111 |  |  |  |  |  |  |  |  | Yes |  |  |  |
| Oribacterium sp. oral taxon 108 | 712414 | 2.24111 |  |  |  |  |  |  |  |  | Yes |  |  |  |
| Oribacterium_u_s | 265975 | 2.24111 |  |  |  |  |  |  |  |  | Yes |  |  |  |
| Oscillibacter sp. ER4 | 1519439 | 2.09135 |  |  |  |  |  |  |  |  | Yes |  |  |  |
| Oscillibacter_u_s | 459786 | 2.09135 |  |  |  |  |  |  |  |  | Yes |  |  |  |
| Pantoea sp. PSNIH1 | 1484158 | 4.86697 |  |  |  |  |  |  |  |  | Yes |  |  |  |
| Pantoea_u_s | 53335 | 4.86697 |  |  |  |  |  |  |  |  | Yes |  |  |  |
| Paracoccus_u_s | 265 | 3.81156 |  |  |  |  |  |  |  |  | Yes |  |  |  |
| Parvimonas sp. oral taxon 110 | 671230 | 1.54191 |  |  |  |  |  |  |  |  | Yes |  |  |  |
| Parvimonas sp. oral taxon 393 | 713008 | 1.54191 |  |  |  |  |  |  |  |  | Yes |  |  |  |
| Parvimonas_u_s | 543311 | 1.54191 |  |  |  |  |  |  |  |  | Yes |  |  |  |
| Patulibacter_u_s | 361607 | 5.02829 |  |  |  |  |  |  |  |  | Yes |  |  |  |
| Pelosinus_u_s | 365348 | 4.31776 |  |  |  |  |  |  |  |  | Yes |  |  |  |
| Peptoclostridium_u_s | 1481960 | 2.56758 |  |  |  |  |  |  |  |  | Yes |  |  |  |
| Peptoniphilus sp. oral taxon 836 | 671216 | 1.67395 |  |  |  |  |  |  |  |  | Yes |  |  |  |
| Peptoniphilus_u_s | 162289 | 1.67395 |  |  |  |  |  |  |  |  | Yes |  |  |  |
| Peptostreptococcaceae bacterium AS15 | 936556 | 2.65464 |  |  |  |  |  |  |  |  | Yes |  |  |  |
| Peptostreptococcaceae bacterium oral taxon 113 | 1321783 | 2.08825 |  |  |  |  |  |  |  |  | Yes |  |  |  |
| Peptostreptococcus sp. MV1 | 1219626 | 2.06349 |  |  |  |  |  |  |  |  | Yes |  |  |  |
| Peptostreptococcus_u_s | 1257 | 2.06349 |  |  |  |  |  |  |  |  | Yes |  |  |  |
| Phascolarctobacterium sp. CAG:207 | 1262914 | 1.75319 |  |  |  |  |  |  |  |  | Yes |  |  |  |
| Phyllobacterium_u_s | 28100 | 4.72925 |  |  |  |  |  |  |  |  | Yes |  |  |  |
| Planococcus_u_s | 1372 | 3.62554 |  |  |  |  |  |  |  |  | Yes |  |  |  |
| Polaribacter_u_s | 52959 | 3.24116 |  |  |  |  |  |  |  |  | Yes |  |  |  |
| Polaromonas_u_s | 52972 | 4.5195 |  |  |  |  |  |  |  |  | Yes |  |  |  |
| Pontibacter_u_s | 323449 | 4.62379 |  |  |  |  |  |  |  |  | Yes |  |  |  |
| Porphyromonas sp. KLE 1280 | 997829 | 2.02909 |  |  |  |  |  |  |  |  | Yes |  |  |  |
| Porphyromonas sp. oral taxon 278 | 712437 | 2.02909 |  |  |  |  |  |  |  |  | Yes |  |  |  |
| Porphyromonas sp. oral taxon 279 | 712438 | 2.02909 |  |  |  |  |  |  |  |  | Yes |  |  |  |
| Porphyromonas_u_s | 836 | 2.02909 |  |  |  |  |  |  |  |  | Yes |  |  |  |
| Prevotella conceptionensis | 340486 | 4.08012 |  |  |  |  |  |  |  |  | Yes |  |  |  |
| Prevotella sp. C561 | 563031 | 2.76159 |  |  |  |  |  |  |  |  | Yes |  |  |  |
| Prevotella sp. F0091 | 1227276 | 2.76159 |  |  |  |  |  |  |  |  | Yes |  |  |  |
| Prevotella sp. HJM029 | 1433844 | 2.76159 |  |  |  |  |  |  |  |  | Yes |  |  |  |
| Prevotella sp. ICM33 | 1161412 | 2.76159 |  |  |  |  |  |  |  |  | Yes |  |  |  |
| Prevotella sp. MSX73 | 1032506 | 2.76159 |  |  |  |  |  |  |  |  | Yes |  |  |  |
| Prevotella sp. oral taxon 299 | 652716 | 2.76159 |  |  |  |  |  |  |  |  | Yes |  |  |  |
| Prevotella sp. oral taxon 306 | 712461 | 2.76159 |  |  |  |  |  |  |  |  | Yes |  |  |  |
| Prevotella sp. oral taxon 317 | 652721 | 2.76159 |  |  |  |  |  |  |  |  | Yes |  |  |  |
| Prevotella sp. oral taxon 472 | 655809 | 2.76159 |  |  |  |  |  |  |  |  | Yes |  |  |  |
| Prevotella sp. oral taxon 473 | 712469 | 2.76159 |  |  |  |  |  |  |  |  | Yes |  |  |  |
| Prevotella_u_s | 838 | 2.76159 |  |  |  |  |  |  |  |  | Yes |  |  |  |
| Promicromonospora_u_s | 43676 | 6.40908 |  |  |  |  |  |  |  |  | Yes |  |  |  |
| Propionibacterium sp. 434-HC2 | 936048 | 1.9999 |  |  |  |  |  |  |  |  | Yes |  |  |  |
| Propionibacterium sp. 5_U_42AFAA | 450748 | 1.9999 |  |  |  |  |  |  |  |  | Yes |  |  |  |
| Propionibacterium sp. HGH0353 | 1203571 | 1.9999 |  |  |  |  |  |  |  |  | Yes |  |  |  |
| Propionibacterium sp. KPL1844 | 1203573 | 1.9999 |  |  |  |  |  |  |  |  | Yes |  |  |  |
| Propionibacterium sp. KPL2009 | 1203635 | 1.9999 |  |  |  |  |  |  |  |  | Yes |  |  |  |
| Propionibacterium sp. oral taxon 192 | 671222 | 1.9999 |  |  |  |  |  |  |  |  | Yes |  |  |  |
| Propionibacterium_u_s | 1743 | 1.9999 |  |  |  |  |  |  |  |  | Yes |  |  |  |
| Pseudomonas sp. Ag1 | 1197727 | 6.178 |  |  |  |  |  |  |  |  | Yes |  |  |  |
| Pseudomonas sp. CBZ-4 | 1163065 | 6.178 |  |  |  |  |  |  |  |  | Yes |  |  |  |
| Pseudomonas_u_s | 286 | 6.178 |  |  |  |  |  |  |  |  | Yes |  |  |  |
| Pseudonocardia_u_s | 1847 | 6.52738 |  |  |  |  |  |  |  |  | Yes |  |  |  |
| Pseudoxanthomonas_u_s | 83618 | 4.3851 |  |  |  |  |  |  |  |  | Yes |  |  |  |
| Psychrobacter_u_s | 497 | 3.12006 |  |  |  |  |  |  |  |  | Yes |  |  |  |
| Psychroflexus_u_s | 83612 | 2.65126 |  |  |  |  |  |  |  |  | Yes |  |  |  |
| Pusillimonas_u_s | 305976 | 3.30136 |  |  |  |  |  |  |  |  | Yes |  |  |  |
| Ralstonia_u_s | 48736 | 5.36575 |  |  |  |  |  |  |  |  | Yes |  |  |  |
| Rheinheimera_u_s | 67575 | 4.15261 |  |  |  |  |  |  |  |  | Yes |  |  |  |
| Rhodopirellula_u_s | 265488 | 6.55587 |  |  |  |  |  |  |  |  | Yes |  |  |  |
| Rhodopseudomonas_u_s | 1073 | 5.40495 |  |  |  |  |  |  |  |  | Yes |  |  |  |
| Roseiflexus_u_s | 120961 | 5.8016 |  |  |  |  |  |  |  |  | Yes |  |  |  |
| Roseomonas_u_s | 125216 | 5.80294 |  |  |  |  |  |  |  |  | Yes |  |  |  |
| Ruminococcus sp. 5_1_39BFAA | 457412 | 2.11841 |  |  |  |  |  |  |  |  | Yes |  |  |  |
| Runella_u_s | 105 | 7.13646 |  |  |  |  |  |  |  |  | Yes |  |  |  |
| Saccharopolyspora_u_s | 1835 | 6.33064 |  |  |  |  |  |  |  |  | Yes |  |  |  |
| Saccharothrix_u_s | 2071 | 9.304 |  |  |  |  |  |  |  |  | Yes |  |  |  |
| Salinispora_u_s | 168694 | 5.45102 |  |  |  |  |  |  |  |  | Yes |  |  |  |
| Sandarakinorhabdus_u_s | 362865 | 3.19859 |  |  |  |  |  |  |  |  | Yes |  |  |  |
| Scardovia_u_s | 196081 | 1.50837 |  |  |  |  |  |  |  |  | Yes |  |  |  |
| Sedimentibacter sp. B4 | 304766 | 3.61902 |  |  |  |  |  |  |  |  | Yes |  |  |  |
| Segniliparus_u_s | 286801 | 3.37258 |  |  |  |  |  |  |  |  | Yes |  |  |  |
| Selenomonas sp. CM52 | 936381 | 2.55831 |  |  |  |  |  |  |  |  | Yes |  |  |  |
| Selenomonas sp. F0473 | 999423 | 2.55831 |  |  |  |  |  |  |  |  | Yes |  |  |  |
| Selenomonas sp. FOBRC6 | 936572 | 2.55831 |  |  |  |  |  |  |  |  | Yes |  |  |  |
| Selenomonas sp. FOBRC9 | 936573 | 2.55831 |  |  |  |  |  |  |  |  | Yes |  |  |  |
| Selenomonas sp. oral taxon 137 | 712531 | 2.55831 |  |  |  |  |  |  |  |  | Yes |  |  |  |
| Selenomonas sp. oral taxon 138 | 712532 | 2.55831 |  |  |  |  |  |  |  |  | Yes |  |  |  |
| Selenomonas sp. oral taxon 149 | 712535 | 2.55831 |  |  |  |  |  |  |  |  | Yes |  |  |  |
| Selenomonas sp. oral taxon 892 | 1321785 | 2.55831 |  |  |  |  |  |  |  |  | Yes |  |  |  |
| Selenomonas_u_s | 970 | 2.55831 |  |  |  |  |  |  |  |  | Yes |  |  |  |
| Serinicoccus_u_s | 265976 | 3.52102 |  |  |  |  |  |  |  |  | Yes |  |  |  |
| Serratia_u_s | 613 | 5.30839 |  |  |  |  |  |  |  |  | Yes |  |  |  |
| Shewanella_u_s | 22 | 4.86107 |  |  |  |  |  |  |  |  | Yes |  |  |  |
| Shuttleworthia sp. MSX8B | 936574 | 2.10262 |  |  |  |  |  |  |  |  | Yes |  |  |  |
| Shuttleworthia_u_s | 177971 | 2.10262 |  |  |  |  |  |  |  |  | Yes |  |  |  |
| Slackia sp. CM382 | 1111137 | 2.0414 |  |  |  |  |  |  |  |  | Yes |  |  |  |
| Solirubrobacter_u_s | 207599 | 5.86208 |  |  |  |  |  |  |  |  | Yes |  |  |  |
| Sphingomonas sp. Ant H11 | 1564113 | 4.04124 |  |  |  |  |  |  |  |  | Yes |  |  |  |
| Sphingomonas_u_s | 13687 | 4.04124 |  |  |  |  |  |  |  |  | Yes |  |  |  |
| Sphingopyxis_u_s | 165697 | 4.30741 |  |  |  |  |  |  |  |  | Yes |  |  |  |
| Spirosoma_u_s | 107 | 8.99194 |  |  |  |  |  |  |  |  | Yes |  |  |  |
| Spongiibacter_u_s | 630749 | 3.40738 |  |  |  |  |  |  |  |  | Yes |  |  |  |
| Staphylococcus sp. DORA_6_22 | 1403935 | 2.53498 |  |  |  |  |  |  |  |  | Yes |  |  |  |
| Staphylococcus sp. M0480 | 1388318 | 2.53498 |  |  |  |  |  |  |  |  | Yes |  |  |  |
| Staphylococcus sp. MDS7B | 1209359 | 2.53498 |  |  |  |  |  |  |  |  | Yes |  |  |  |
| Staphylococcus_u_s | 1279 | 2.53498 |  |  |  |  |  |  |  |  | Yes |  |  |  |
| Stenotrophomonas sp. SKA14 | 391601 | 4.43876 |  |  |  |  |  |  |  |  | Yes |  |  |  |
| Stenotrophomonas_u_s | 40323 | 4.43876 |  |  |  |  |  |  |  |  | Yes |  |  |  |
| Streptococcus sp. 2_1_36FAA | 469609 | 2.03679 |  |  |  |  |  |  |  |  | Yes |  |  |  |
| Streptococcus sp. ACS2 | 936576 | 2.03679 |  |  |  |  |  |  |  |  | Yes |  |  |  |
| Streptococcus sp. AS14 | 936577 | 2.03679 |  |  |  |  |  |  |  |  | Yes |  |  |  |
| Streptococcus sp. AS20 | 936578 | 2.03679 |  |  |  |  |  |  |  |  | Yes |  |  |  |
| Streptococcus sp. BS21 | 1161414 | 2.03679 |  |  |  |  |  |  |  |  | Yes |  |  |  |
| Streptococcus sp. BS35b | 1105032 | 2.03679 |  |  |  |  |  |  |  |  | Yes |  |  |  |
| Streptococcus sp. C300 | 563036 | 2.03679 |  |  |  |  |  |  |  |  | Yes |  |  |  |
| Streptococcus sp. CM6 | 936580 | 2.03679 |  |  |  |  |  |  |  |  | Yes |  |  |  |
| Streptococcus sp. DBCMS | 1604598 | 2.03679 |  |  |  |  |  |  |  |  | Yes |  |  |  |
| Streptococcus sp. DORA_10 | 1403937 | 2.03679 |  |  |  |  |  |  |  |  | Yes |  |  |  |
| Streptococcus sp. F0441 | 999424 | 2.03679 |  |  |  |  |  |  |  |  | Yes |  |  |  |
| Streptococcus sp. GMD3S | 1169672 | 2.03679 |  |  |  |  |  |  |  |  | Yes |  |  |  |
| Streptococcus sp. GMD5S | 1169674 | 2.03679 |  |  |  |  |  |  |  |  | Yes |  |  |  |
| Streptococcus sp. HSISM1 | 1316408 | 2.03679 |  |  |  |  |  |  |  |  | Yes |  |  |  |
| Streptococcus sp. HSISS2 | 1316411 | 2.03679 |  |  |  |  |  |  |  |  | Yes |  |  |  |
| Streptococcus sp. I-G2 | 1156431 | 2.03679 |  |  |  |  |  |  |  |  | Yes |  |  |  |
| Streptococcus sp. I-P16 | 1156433 | 2.03679 |  |  |  |  |  |  |  |  | Yes |  |  |  |
| Streptococcus sp. M143 | 563037 | 2.03679 |  |  |  |  |  |  |  |  | Yes |  |  |  |
| Streptococcus sp. M334 | 563038 | 2.03679 |  |  |  |  |  |  |  |  | Yes |  |  |  |
| Streptococcus sp. OBRC6 | 936587 | 2.03679 |  |  |  |  |  |  |  |  | Yes |  |  |  |
| Streptococcus sp. oral taxon 056 | 712620 | 2.03679 |  |  |  |  |  |  |  |  | Yes |  |  |  |
| Streptococcus sp. oral taxon 058 | 712622 | 2.03679 |  |  |  |  |  |  |  |  | Yes |  |  |  |
| Streptococcus sp. oral taxon 071 | 712630 | 2.03679 |  |  |  |  |  |  |  |  | Yes |  |  |  |
| Streptococcus sp. SR1 | 1161416 | 2.03679 |  |  |  |  |  |  |  |  | Yes |  |  |  |
| Streptococcus sp. SR4 | 1161417 | 2.03679 |  |  |  |  |  |  |  |  | Yes |  |  |  |
| Streptococcus sp. VT 162 | 1419814 | 2.03679 |  |  |  |  |  |  |  |  | Yes |  |  |  |
| Streptococcus_u_s | 1301 | 2.03679 |  |  |  |  |  |  |  |  | Yes |  |  |  |
| Streptomyces_u_s | 1883 | 8.21993 |  |  |  |  |  |  |  |  | Yes |  |  |  |
| Subdoligranulum_u_s | 292632 | 2.3723 |  |  |  |  |  |  |  |  | Yes |  |  |  |
| Sulfobacillus_u_s | 28033 | 3.35869 |  |  |  |  |  |  |  |  | Yes |  |  |  |
| Sulfuricurvum_u_s | 286130 | 2.01346 |  |  |  |  |  |  |  |  | Yes |  |  |  |
| Sutterella_u_s | 40544 | 1.83662 |  |  |  |  |  |  |  |  | Yes |  |  |  |
| Synechocystis sp. PCC 7509 | 927677 | 3.65994 |  |  |  |  |  |  |  |  | Yes |  |  |  |
| Tannerella sp. oral taxon HOT-286 | 712710 | 2.97354 |  |  |  |  |  |  |  |  | Yes |  |  |  |
| Tatumella_u_s | 82986 | 3.51785 |  |  |  |  |  |  |  |  | Yes |  |  |  |
| Taylorella_u_s | 29574 | 1.64293 |  |  |  |  |  |  |  |  | Yes |  |  |  |
| Thalassospira_u_s | 168934 | 4.3678 |  |  |  |  |  |  |  |  | Yes |  |  |  |
| Thauera_u_s | 33057 | 3.72539 |  |  |  |  |  |  |  |  | Yes |  |  |  |
| Thermanaerovibrio_u_s | 81461 | 1.86466 |  |  |  |  |  |  |  |  | Yes |  |  |  |
| Thermoactinomyces_u_s | 2023 | 2.62331 |  |  |  |  |  |  |  |  | Yes |  |  |  |
| Thermoanaerobacterium_u_s | 28895 | 2.9797 |  |  |  |  |  |  |  |  | Yes |  |  |  |
| Thermodesulfatator_u_s | 241192 | 1.28293 |  |  |  |  |  |  |  |  | Yes |  |  |  |
| Thermodesulfobacterium_u_s | 1740 | 1.56156 |  |  |  |  |  |  |  |  | Yes |  |  |  |
| Thioalkalivibrio_u_s | 106633 | 2.82028 |  |  |  |  |  |  |  |  | Yes |  |  |  |
| Thiomicrorhabdus_u_s | 2039723 | 2.6504 |  |  |  |  |  |  |  |  | Yes |  |  |  |
| Thiomonas_u_s | 32012 | 3.60942 |  |  |  |  |  |  |  |  | Yes |  |  |  |
| Treponema sp. OMZ 838 | 1539298 | 2.35177 |  |  |  |  |  |  |  |  | Yes |  |  |  |
| Treponema_u_s | 157 | 2.35177 |  |  |  |  |  |  |  |  | Yes |  |  |  |
| Tsukamurella_u_s | 2060 | 4.5034 |  |  |  |  |  |  |  |  | Yes |  |  |  |
| Variovorax_u_s | 34072 | 7.00465 |  |  |  |  |  |  |  |  | Yes |  |  |  |
| Veillonella sp. 3_1_44 | 457416 | 1.93796 |  |  |  |  |  |  |  |  | Yes |  |  |  |
| Veillonella sp. 6_1_27 | 450749 | 1.93796 |  |  |  |  |  |  |  |  | Yes |  |  |  |
| Veillonella sp. DORA_B_18_19_23 | 1403933 | 1.93796 |  |  |  |  |  |  |  |  | Yes |  |  |  |
| Veillonella sp. oral taxon 158 | 671228 | 1.93796 |  |  |  |  |  |  |  |  | Yes |  |  |  |
| Veillonella_u_s | 29465 | 1.93796 |  |  |  |  |  |  |  |  | Yes |  |  |  |
| Verminephrobacter_u_s | 364316 | 4.42226 |  |  |  |  |  |  |  |  | Yes |  |  |  |
| Xanthomonas_u_s | 338 | 4.85006 |  |  |  |  |  |  |  |  | Yes |  |  |  |
| Yersinia_u_s | 629 | 4.64328 |  |  |  |  |  |  |  |  | Yes |  |  |  |
